# Bibliometrics of Functional Polymeric Biomaterials with Bioactive Properties Prepared by Radiation-Induced Graft Copolymerisation: A Review

**DOI:** 10.3390/polym14224831

**Published:** 2022-11-10

**Authors:** Mostafa Yusefi, Mohamed Mahmoud Nasef, Mohammad Ali Tareq, Bhuvanesh Gupta, Kamyar Shameli, Roshafima Rasit Ali, Teo Ming Ting, Hesham Ali El Enshasy

**Affiliations:** 1 Department of Chemical and Environmental Engineering, Malaysia-Japan International Institute of Technology, Universiti Teknologi Malaysia, Kuala Lumpur 54100, Malaysia; 2 Advanced Materials Research Group, Center of Hydrogen Energy, Universiti Teknologi Malaysia, Jalan Sultan Yahya Putra, Kuala Lumpur 54100, Malaysia; 3 Department of Management of Technology, Malaysia-Japan International Institute of Technology, Universiti Teknologi Malaysia, Kuala Lumpur 54100, Malaysia; 4 Bioengineering Laboratory, Department of Textile Technology, Indian Institute of Technology, New Delhi 110016, India; 5 Radiation Processing Technology Division, Malaysian Nuclear Agency, Kajang 43000, Selangor, Malaysia; 6 Institute of Bioproduct Development (IBD), Universiti Teknologi Malaysia (UTM), Skudai, Johor Bahru 81310, Johor, Malaysia; 7 City of Scientific Research and Technology Applications (SRTA), New Burg Al Arab, Alexandria 21934, Egypt

**Keywords:** bibliometric analysis, functional polymeric biomaterials, radiation induced grafting, medical and biomedical applications, enzyme carriers, antimicrobial fabrics

## Abstract

Functional polymeric biomaterials (FPBMs) with bioactive characteristics obtained by radiation-induced graft copolymerisation (RIGC) have been subjected to intensive research and developed into many commercial products. Various studies have reported the development of a variety of radiation-grafted FPBMs. However, no reports dealing with the quantitative evaluations of these studies from a global bibliographic perspective have been published. Such bibliographic analysis can provide information to overcome the limitations of the databases and identify the main research trends, together with challenges and future directions. This review aims to provide an unprecedented bibliometric analysis of the published literature on the use of RIGC for the preparation of FPBMs and their applications in medical, biomedical, biotechnological, and health care fields. A total of 235 publications obtained from the Web of Science (WoS) in the period of 1985–2021 were retrieved, screened, and evaluated. The records were used to manifest the contributions to each field and underline not only the top authors, journals, citations, years of publication, and countries but also to highlight the core research topics and the hubs for research excellence on these materials. The obtained data overviews are likely to provide guides to early-career scientists and their research institutions and promote the development of new, timely needed radiation-grafted FPBMs, in addition to extending their applications.

## 1. Introduction

Functional polymeric biomaterials (FPBMs) are a class of materials that is receiving increasing interest because of their relevance to applications in various areas impacting human life and living [1]. FPBMs represent the most popular usage of natural, synthetic, or hybrid polymeric materials interacting with biological regimes and are used to protect against microbes and regenerate, repair, and treat any type of tissue in the organs, or improve the functions of the human body [2]. Thus, the research on FPBMs has enabled the development of various implants, medical devices, drug carriers, and scaffolds for tissue engineering with greater antimicrobial resistance, biocompatibility, and biofunctionality together with lower cytotoxicity against malignant cells [3]. FPBMs can be developed using various methods involving surface treatment such as etching, metallisation, ion sputtering, chemical grafting, and radiation-induced graft copolymerisation (RIGC) with low-energy radiation sources (plasma, laser treatment, and UV lamp) and high-energy radiation sources such as γ-rays and electron beam (EB) [4]. Each modification method has its pros and cons, and the selection of a particular method usually controls the developed topical structure and the level as well as the stability of the enhanced properties conferred to the polymer substrates.

Of all methods, RIGC is a distinctive technique for modification of polymer substrates that has been known for the past six decades. The versatility of this method is derived from its ability to permanently modify polymeric substrates by imparting new functionalities originated for the incoming polar monomers (acrylic or vinylic monomers) without compromising the inherent properties of the parent polymers. Such versatility enabled this technology to be used to develop many hybrid polymeric materials with desired properties [5,6,7,8]. The advantages of this method over its well-established chemically induced counterpart are not only in its ability to meticulously control the grafting yield by manipulation of the grafting parameters and the absence of detrimental impurities that maintain the purity of the product but also in the consumption of fewer chemicals and the ability to overcome product shaping problems. This is because grafting reactions can be started with polymer substrates of different physical forms, including films, non-woven fabric, particles, and fibres. Moreover, RIGC is also capable of achieving bulk modifications in the substrates when using γ-rays and EB sources, with the capability to scale up grafted products compared to plasma- and UV-induced grafting, which are limited to laboratory scale [9,10].

**Figure 1:**
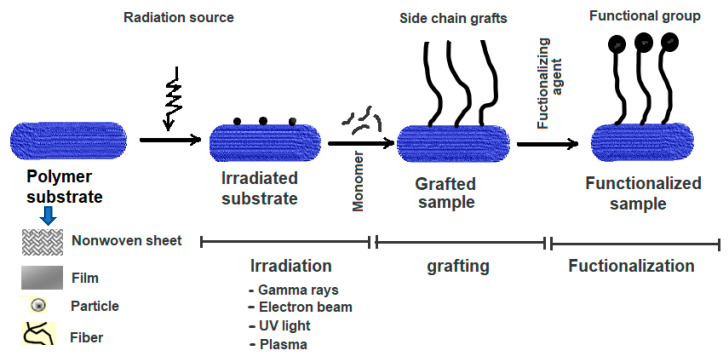
Schematic representation for preparation of functional polymeric materials by RIGC and functionalisation.

RIGC works by exposing a polymer substrate to ionising radiation, leading to the generation of active sites or radicals. The formed radicals initiate copolymerisation when exposed to monomer molecules, forming macroradicals that allow propagation of the molecular chains to form side chain grafts when terminated. RIGC can be carried out by the direct interaction between the monomer and the polymer substrate during irradiation, and this method is called simultaneous radiation grafting. Alternatively, the polymer substrate is initially irradiated and subsequently brought in contact with the monomer molecules in a separate step, and the technique is called pre-irradiation grafting. Introducing functional groups can be made by direct grafting of functional (acrylic) monomers or by a post-grafting reaction upon grafting vinylic monomers. Figure 1 shows a schematic diagram representing the preparation of functional polymeric materials by irradiation, grafting of the monomer, and subsequent functionalisation. More details on RIGC methods, including their advantages and disadvantages, can be found elsewhere [11].

RIGC has been extensively used to endow interesting functional characteristics such as hydrophilicity, hydrophobicity, ion conduction, metal ion binding capacity, adhesion, microbial and fouling resistance, and biocompatibility to different polymer substrates. This led to the development of various functional polymeric materials through the integration of various monomer/polymer combinations enduring desired characteristics to polymer substrates. The resultant radiation-grafted functional materials’ applications include battery separators [12,13,14], polymer electrolyte membranes [15,16], chelating adsorbents [17,18,19], ion exchange membranes [20,21], and FPBMs [6,22].

**Figure 2:**
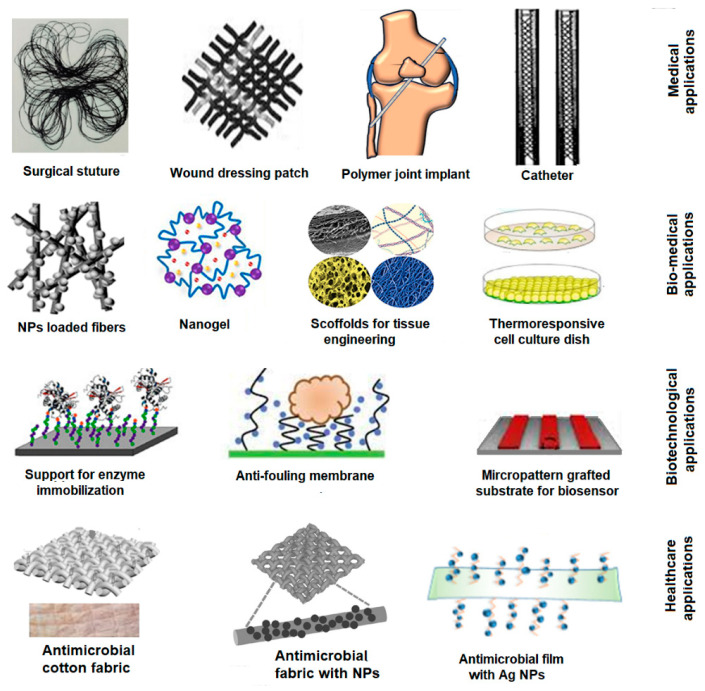
Schematic diagram of examples of FPBMs prepared by RIGC for various applications.

Particularly, the application of RIGC for the development of FPBMs is one of the areas that has received paramount efforts since the early days of the technique’s invention by A. Chapiro (a French scientist) [23] and A. Charlesby (a British scientist) [24]. Subsequently, many researchers used this technique to make enormous contributions to designing and developing innovative materials for medical, biomedical, health care, and biotechnological applications. Figure 2 shows some examples of FPBMs prepared by RIGC for various applications. This leads to the emergence of many potential radiation-grafted materials with great potential for commercialisation, and this field seems to be broadly opening for imaginative future developments.

Several articles have been devoted to reviewing the use of RIGC for the development of adsorbents for purifications and separations in environmental applications, including wastewater treatment and CO_2_ capture [25,26], polymer electrolyte membranes and separators for energy conversion and storage [9,10,27], and protein capture [21]. The preparation and applications of biomaterials such as stimuli-responsive polymer systems and their polymer-biomolecule (protein) conjugates were also reviewed on two occasions [28,29]. Likewise, the use of RIGC or crosslinking to produce hydrogels of various size ranges (microgels and nanogels) and their applications in various medical and biomedical applications were extensively reviewed in many publications [30,31,32,33,34] with some attention recently paid to reviewing current approaches for crosslinking polysaccharides for hydrogel formation [35]. On the other hand, FPBMs obtained by RIGC have also been reviewed on various occasions, but with the focus limited to biomedical applications [6,26,27,28,29], cell sheet engineering, and the characteristics of thermo-responsive scaffolds [36,37]. Most of the published reviews mainly discussed strategies to construct a variety of radiation-grafted biomaterials with desired structure, properties, dynamic functionality, and biological complexity, taking the target application into account and elaborating on the challenges to the endurance of the imparted functionality. Nevertheless, there is no review reported to evaluate the accumulated publication of the research articles on radiation-grafted FPBMs and their broadly tested applications in the fields of medical, biomedical, biotechnological, and healthcare applications from a global bibliometric perspective.

Bibliometric analysis is a scientific computer-assisted review based on statistical methodology that allows the evaluation of a large amount of written research publications (articles and books) related to given academic topics [38]. This tool grants quantitative analysis of citations in databases such as the Web of Science (WoS) and Scopus to establish not only inter-relationships and impacts of publications in research area but also allows for the efficient identification of core research, influential studies, authors, journals, years of funding organisations, and countries as well as their relationship [39]. The benefit of bibliometric analysis makes it useful for assessing research trends in a variety of fields and the quality of research output, overcoming the limitations of databases, and identifying the factors required for developing new research projects in a time-effective manner [40,41]. This would certainly provide valuable assistance to the research triangle involving researchers, funders, and policymakers.

Bibliometric analysis studies covering various materials-related fields, such as polymer composites for energy storage [42], antibacterial dental adhesive [43], artificial extracellular matrices based on tissue engineering technology [44], liquid membranes [45], enzyme immobilisation [40,46], and chitosan coating for fruits [41], have appeared in the literature recently. However, there have been no bibliometric reviews of radiation-grafted materials and their applications in various fields.

The objective of this article is to report a systematic bibliometric analysis for evaluating the literature published on radiation-grafted FPBMs and their applications in medical, biomedical, biotechnological, and healthcare fields. The bibliometric analysis was carried out based on keywords, co-citations, and performance. The scope of the data mapping included citation analysis and literature distributions with respect to progress in the field of application of radiation-grafted bioactive materials, journals, authors, institutions, and countries in terms of their research output in the period of 1985–2021. The data mapping used various visualisation software such as VOSViewer and CiteSpace to present the information.

## 2. Data Collection and Methods

**Figure 3:**
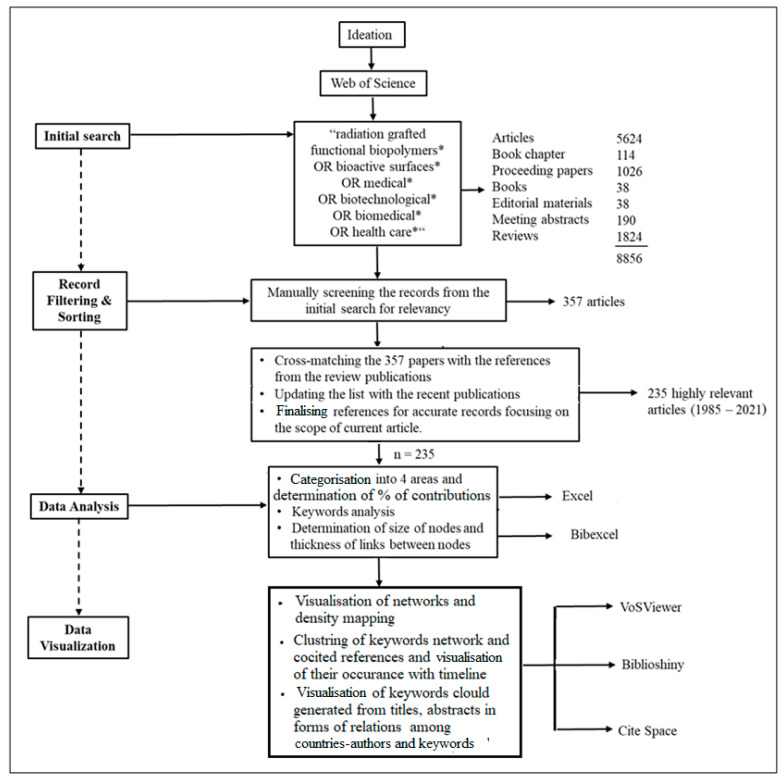
Multi-step strategy used to evaluate the bibliometrics of FPBMs prepared by RIGC.

The initial data mining was carried out between 15 and 22 August 2021 using the WoS database. The principal theme was publications containing the query keywords “radiation grafted functional biopolymers *, OR bioactive surfaces *, OR medical *, OR biotechnological *, OR biomedical *, OR health care *” in the titles or in the abstracts. A total of 8854 records were found, including 5624 articles, 114 book chapters, 1026 proceeding papers, 38 books, 38 editorial materials, 190 meeting abstracts, and 1824 reviews. The noise in the records was high as the basic search was carried out using the booleans “OR” and Field Tag “TS = Topic”. Titles, abstracts, and keywords of the 8854 records were individually checked, and 357 records were identified as related to our interest. Later, these records were carefully screened by reading each paper, taking into consideration the relevance and scope of the topic of interest, and finally 235 papers linked to the investigated applications were finalised for the period of 1985–2021. These 235 articles were searched on the WOS database for extracting the full bibliometric records needed for scientometric analysis. The metadata on the selected 235 articles on radiation-grafted FPBMs was transferred into Microsoft Excel (for Windows 10), BibTex (version 0.99d), and plain text formats suitable for bibliometric analysis using BibExcel (version 2016-02-20) and for visualisation using Biblioshiny (RStudio) (version 3.6.1), VOSViewer (version 1.6.15), and CiteSpace (version 5.3.R9). Particularly, BibExcel was used to conduct the descriptive analysis on the sample dataset once the data were checked for duplication and missing information using Microsoft Excel. The Biblioshiny (RStudio), VOSViewer, and CiteSpace were used for the network and other visualisations for the analyses of citations and references, which supports several types of bibliometrics meeting the needs of visual analytic tasks to depict a systematic review visually. The open-source package Biblioshiny is used in the R language environment, where bibliometrics allowed the completion of the process of the scientific literature analysis and data processing [47]. Biblioshiny, on the other hand, was used to perform pertinent bibliometric and visual analysis based on an interactive web interface. CiteSpace and VOSViewer were used to create the co-occurrence of the keyword network and cluster network. VOSviewer was used to obtain and visualise bibliometric records with regards to popular keywords, citation records, and publication and collaboration networks as reported elsewhere [48]. The bibliometric analysis focuses on the present status, whereas the content analysis helps to describe the structural framework of the literature on radiation-grafted FPBMs. Figure 3 illustrates the multi-step strategy used to evaluate the bibliometrics of FPBMs by RIGC.

The collected 235 references were further sorted out manually based on the specific applications of FPBMs prepared by RIGC that were categorised into four main areas: medical, biomedical, biotechnological, and healthcare applications. Each of these areas was divided into a few classes based on the reported applications. The contribution percentage of each class of applications was estimated by considering the ratio of the number of publications in each class and the total number of publications on FPBMs. The overall classifications were tabulated with their relevant references, and the class percentage data was graphically presented. The applications, which have very rare publications, such as gene delivery [49,50], were ignored, although they might be appealing for preparation with other methods falling beyond the scope of this article.

The keyword analysis network that was carried out to observe the changes in the research themes was sorted out from the cloud generated from the titles and abstracts of the collected sample of the articles. The network visualisation and density mapping of the co-occurrence of the keywords related to the radiation-grafted FPBMs were presented in four interrelated groups. The size of the nodes represents the frequency of the keywords, and the thickness of the links between two nodes represents the co-occurrence of the keywords. The keyword network of publications and co-cited references was clustered, and their co-occurrence was visualised, taking the timeline into consideration. The network was clearly defined, and we considered the co-citation clusters. The clusters were labelled by the index terms selected from the citation frequencies that were arranged vertically in the descending order of the cluster size, and the curves represent the co-citation links between clusters.

## 3. Results and Discussion

### 3.1. Classification and Applications of FPBMs Prepared by RIGC

The design of FPBMs by RIGC was found to focus on the broad aspects of polymer surface modifications, inculcating antimicrobial resistance, biofilm formation prevention, biocompatibility, cytotoxicity resistance, bio-functionality, control-release of therapeutic agents in drug delivery, and healing functionalities in tissue engineering and regenerative medicine [2]. The advantages of this method for the preparation of FPBMs include the use of high-energy radiation for clean initiation (without chemical initiators) and the ability to obtain sterilised products [51]. Applications of FPBMs prepared by RIGC have been frequently reported in the literature, which prevailed the emergence of many urgently needed innovative materials capable of meeting the requirements of diversified applications [6]. Particularly, radiation-grafted FPBMs, such as drug release carriers [52,53], antimicrobial surgical sutures [54], thermo-responsive cell culture plates [55], scaffolds for tissue engineering [56], water-absorbing polymers [57], enzyme carriers [58], antimicrobial catheters [59], implants [60], antimicrobial gauze [61], antimicrobial fabrics [62,63], antibacterial food packing films [64,65], and antifouling membranes [66], have been widely investigated to design efficient, safe, and viable products. Such a wide spectrum of applications emphasises a need for a deep understanding of the knowledge underlying not only radiation chemistry and radiation processing of polymers but also biomedicine, biochemistry, and biomaterials characteristics. The various applications of FPBMs can be classified into four categories, including: (i) medicine, (ii) biomedicine, (iii) biotechnology, and (iv) healthcare, as shown in Table 1.

**Figure 4:**
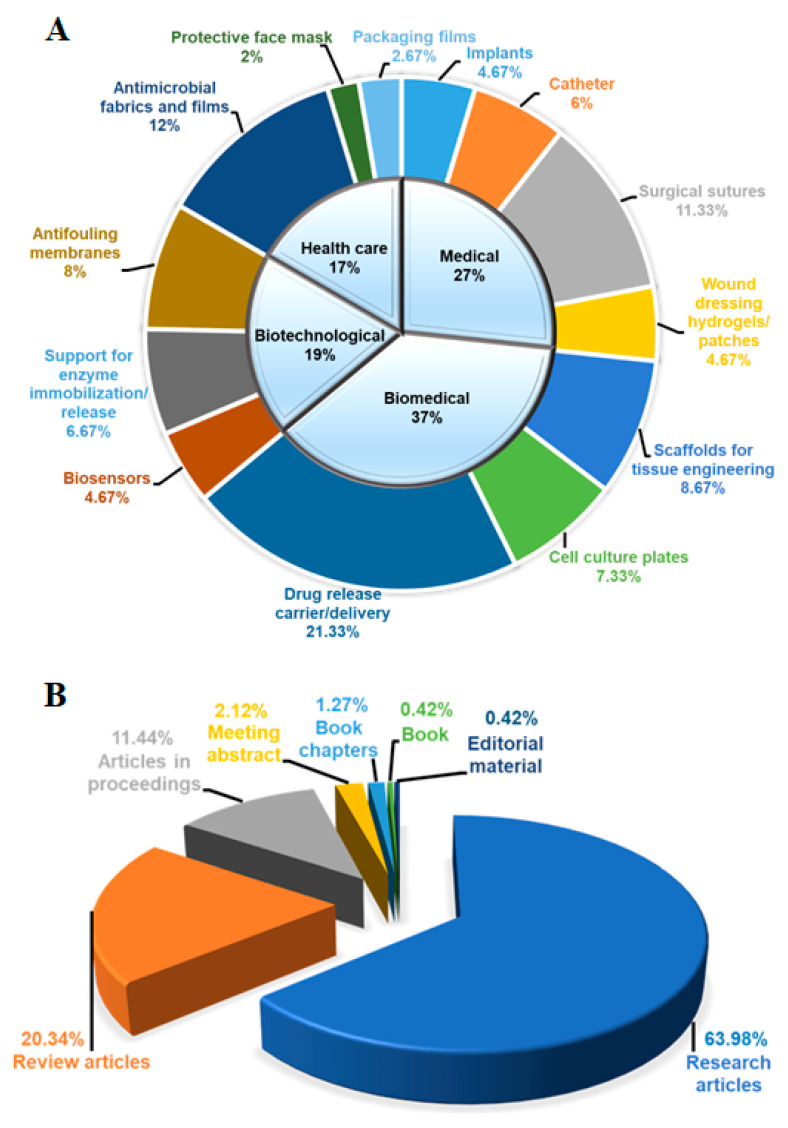
Graphical representation of: (**A**) various applications % of FPBMs with bioactive properties prepared by RIGC and (**B**) distribution % of their related publications. Total number of records is 235.

The percentage of each category and class relative to the total number of articles, together with the distribution of publication on FPBMs prepared by RIGC, are illustrated in Figure 4. It can be observed from Figure 4A that the biomedical field occupied the highest number of applications with 37% of the total publications, followed by 27% for medical, 19% for biotechnological, and 17% for healthcare applications. On the other hand, the percentage of individual applications with respect to the overall publications revealed that the drug carrier/release is the most investigated application with 21.33%, followed by antimicrobial fabrics and films (12%), surgical sutures (11.33%), scaffolds for tissue engineering (8.67%), antifouling membranes (8%), cell culture plates (7.33%), supports for enzyme immobilisation and release (6.67%), catheters (6%), implants (4.67%), biosensors (4.67%), packing films (2.67%), and protective face masks (2%). It is not a surprise that the drug carrier/release application has the highest contribution, and this reflects the significance and wide investigation for such an application since the early days of realising the significance of RIGC. Moreover, this application has unlimited potential to provide drug delivery therapies and devices for many diseases, making such an application a source of innovation and promising technologies. The smallest contribution came from the application protective face masks, which is most likely caused by the high cost of such masks compared with the prices of normal surgical face masks. Nevertheless, the emergence of iodine-containing face masks, which was triggered by the outbreak of the swine flu (H1N1) pandemic in 2009 and commercialised by Ebara Clean Environment Co., Ltd., Japan [63], provides a potential area for further development of new fabrics for masks and coatings with antimicrobial/antiviral properties for combating the outbreak of viruses such as the *Corona* virus.

### 3.2. Distribution of Publications on FPBMs Prepared by RIGC (1985–2021)

A total of 235 records were found to be appropriate after selecting the entire results from the publications on FPBMs prepared by RIGC in the English language literature in the WoS in the period of 1985–2021. As shown in Figure 4B, the entire distribution was classified into eight published sources, which were mainly articles (151 articles = 63.98%) and reviews (48 articles = 20.34%). A total of 186 of these records, representing 78.96%, could be found through the subscribed journals, and the rest were available in their open access counterparts. The average total citation (TC) per the published documents was 164.23, showing the popularity of FPBMs prepared by RIGC for various advanced biomedical demands. The average number of authors per published document was found to be 1.75. The documents related to FPBMs by RIGC were published under various disciplines, including “Physics, Materials, and Chemistry”, followed by “Molecular Biology, and Immunology” and “Medicine, Medical and Clinical Applications”.

### 3.3. Number Annual of Publications on Application of FPBMs Prepared by RIGC since 1985

**Figure 5:**
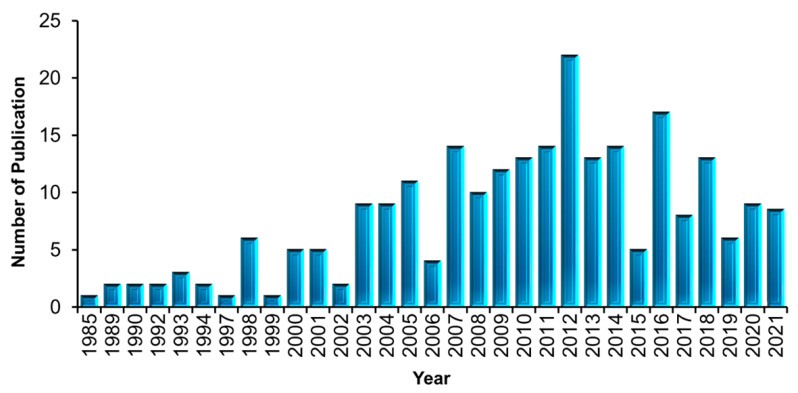
Frequency of publications on FPBMs prepared by RIGC per year in the period of 1985–2021.

Figure 5 indicates the growing trend in FPBMs prepared by RIGC over the last four decades. The number of annual publications on FPBMs prepared by RIGC indicates the presence of an unsteady growth rate. The entire period of 37 years (1985–2021) can be approximately divided into two periods: an initial explorative period until 2002 and a progressive period from 2003 onward. Prior to 2003, the field started to grow slowly, as indicated by the number of documents that were published. Particularly, this period witnessed a growing level of support from the International Atomic Energy Agency (IAEA) to various activities related to the applications of radiation chemistry in biomaterial and bioengineering fields, including three consecutive coordinated research project programmes (CRP) that started in 1983. The first project was on “radiation technology for immobilisation of bioactive materials” in the period of 1983–1987 [193], followed by another project on “radiation processing technology applications in bioengineering” in the period of 1988–1994 [194], and the third one on the “use of radiation processing to prepare biomaterials for applications in medicine” (1996–2000) [195]. This led to a remarkable increase in the number of publications after 2007, in which more than 72% of the studies were published, and the highest annual publication percentage was in 2012 (8.97%). The period of 2009–2021 also witnessed another CRP from the IAEA, focusing on nanoscale radiation engineering of advanced materials for biomedical applications, which involves the use of ionising radiation in the synthesis, modification, and characterisation of nanogels, nanoparticles, nanovehicles, nanoporous membranes, and surfaces with enhanced biocompatibility for potential biomedical applications, such as cell sheet engineering and artificial tissue construction, diagnostics and imaging, and drug delivery [196].

### 3.4. Leading Journals for Publication Related to FPBMs Prepared by RIGC

Table 2 shows the most influential journals associated with the published articles on FPBMs prepared by RIGC, taking into consideration the total publications (TP) and TC. The list of journals is headed by Radiation Physics and Chemistry, followed by the Journal of Applied Polymer Science, ACTA Biomaterialia, Progress in Polymer Science, Biomaterials, Polymer, Macromolecules, Materials and Langmuir. It can be seen that seven out of ten of the most influential journals for publishing articles on the preparation of FPBMs by RIGC are falling in the Q1 quartile index ranking in WoS, with three of these journals being published in the UK, two in the Netherlands, and two in the USA. The sequence of leading publishers is Elsevier (eight journals), the American Chemical Society (two journals), Wiley (one journal), and MDPI (one journal). Of all journals, the journal of Radiation Physics and Chemistry (Elsevier) is ranked first in the number of publications with 28 articles (11.96%) and a TC of 904 despite being the second-lowest in terms of IF (i.e., 2.858 in 2021) in this list. This was followed by the Journal of Applied Polymer Science (Wiley) with an IF of 3.057 (in 2021) with 16 articles (6.83%) and a TC of 856. Interestingly, the Journal of Applied Polymer Science (Wiley) published the first study on FPBMs by RIGC in 1985 [82]. Progress in Polymer Science journal, with the highest IF of 31.28 in 2021, is in the middle of this list with only eight articles (3.41%), albeit with the highest TC of 5506 due to its widely established reputation as an international journal for publishing review articles only. The open-access journal “Materials” from MDPI is the second-last of the listed journals, and this is because it is a new journal in the area of FPBMs prepared by RIGC.

### 3.5. Highly Cited Published Articles

#### Highly Cited Review Articles

Among the search results, many review articles addressing different types of FPBMs prepared by RIGC were published and subjected to analysis in this study, although they might not provide sufficient research progress indications as they only reviewed the progress that was limited to several years before the submission time. Table 3 shows the top 10 highly cited review articles on FPBMs prepared by RIGC. Five review articles that were published in Nature Materials [107], Advanced Materials [197], Seminars in Immunology [198], Progress in Polymer Science [29], and Progress in Polymer Science [199] received the highest citations in the period of 2004–2010 with a TC of 4011, 2689, 2648, 1976, and 1059, respectively. The major contributions in these top five review articles came from the research groups in the US [29,197,198,199]. It can be noticed from the table that half of the top 10 highly cited review articles fall in the open access literature, which is freely accessible worldwide.

### 3.6. Highly Cited Research Articles

Table 4 indicates the top 10 highly cited research articles on FPBMs prepared by RIGC and their relevant applications such as controlled drug delivery systems, cell culture plates, biosensors, antimicrobial surfaces, and implants. The first most-cited research article by Dutch researchers was published in the Macromolecule Journal in 1993 (919 TC) [152]. Okano and co-workers [118] (researchers from Japan) published the second-most-cited article on an essential tissue engineering related to FPBMs prepared by RIGC, which obtained a TC of 792. In this article, the surface of the tissue culture polystyrene dishes was successfully grafted onto a temperature-responsive polymer (poly(N-isopropylacrylamide)) using electron beam irradiation to obtain hydrophilic and hydrophobic surfaces below and above 32 °C, respectively. These excellent temperature responses and thermo-switchable assessments were found to be efficient in delivering localised and controllable cell attachment/detachment without damage. Okano et al. [121] extended their work and published another highly cited two articles (ranked 7 and 10) in 2004 and in 2001, respectively. Both articles focused on temperature-responsive cell culture surfaces/plates with the latter article dedicated to functional cardiac myocyte tissues. This made the work of Okano and co-workers on the cell culture plate among the top cited research in the field of FPBMs prepared by RIGC, which were subsequently commercialised.

The third most-cited research article was written by Rogers and co-workers [204] and was published in Nature Materials with the highest IF (43.84) and C/Y (86.12) in 2013. This research article indicated that the synthesis of microscale temperature sensors was necessary for both precision evaluation and mapping characterisation in a non-invasive procedure onto the skin surface. This device and method offered precise measurement of the skin temperature as affected by skin hydration, mental and physical actions, and vasoconstriction/dilation.

### 3.7. The Top Influential Authors on Development of FPBMs Prepared by RIGC

Table 5 shows the top 10 leading authors who have contributed to FPBMs prepared by the RIGC. These authors’ main research areas primarily fall within chemistry and biomedical engineering, in addition to radiation chemistry and macromolecules. A total of 709 authors contributed to the research on FPBMs prepared by RIGC. Amongst the 10 top co-cited authors, T. Okano (4854 TC) had the highest rank, followed by M. Yamato (2855 TC), A. Kikuchi (2075 TC), E. Bucio (592 TC), and B. Gupta (365 TC). It can be noticed from the table that all three Japanese researchers have the highest TC. On the one hand, E. Bucio, with the highest number of publications (30), had only 592 TC. A. Kikuchi, with only seven publications, had prominently received a total of 2075 TC. On the other hand, T. Okano can be considered the most influential author in terms of both publication (20) and TC (4854). It can be noted that T. Okano has a longer history of publications on FPBMs prepared by RIGC (1990–2018) compared to other authors such as H. Singh, who published his research articles in the period 1989–2008. Thus, he can be considered the most influential author in the area of FPBMs prepared by RIGC. Finally, it was interestingly found that the top 10 authors not only came from the same country but also had the same affiliations, suggesting that the key to success for such authors was working in a team in addition to the availability of funds and research facilities.

**Figure 6:**
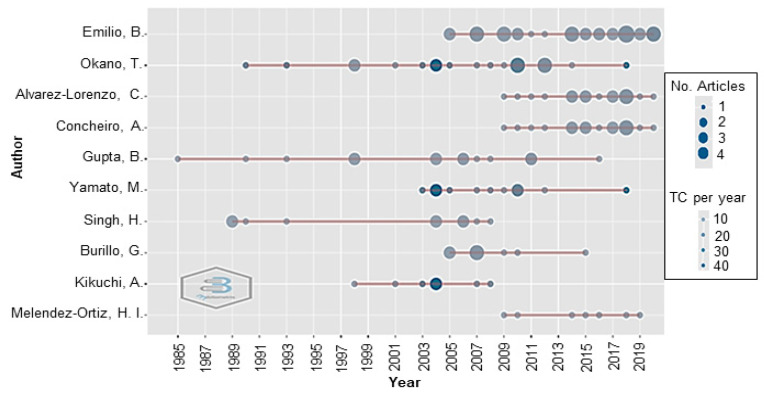
Top 10 authors’ productivity over the period of 1985–2021. The line represents an author’s timeline; the size of the bubbles is proportional to the number of documents produced by an author per year; the colour intensity of the bubble is proportional to the total number of citations per year; the first bubble on the line indicates when the author began to publish in the field; the bigger the bubble is, the higher the number of articles published by an author per year; bubbles with deeper colour intensity indicate higher citation counts.

Results from the top 10 most prolific authors in the field of RIGC from 1985 to 2021 based on the dataset are presented in Figure 6, which shows the top 10 authors with high productivity over the years. These authors have shown consistency in their contributions to the research body in this field. The top authors were found to be more active in the period of 2009–2019 based on TP and the h-index, which is calculated by counting the number of publications for which an author has been cited by other authors at least that same number of times. B. Gupta (h-index: 12, TP: 12) was found to have the longest impact in this research area, followed by T. Okano (h-index: 19, TP: 20). B. Emilio (h-index: 16, TP: 30) has the highest number of publications in this research area, even though his first publication was in 2005. Both H. Alvarez-Loranzo (h-index: 11, TP: 16) and A. Concherio (h-index: 11, TP: 16) have co-authored several papers in this area since 2009.

### 3.8. The Most Influential Countries to Provide Funding for FPBMs Prepared by RIGC

The leading countries in providing funds for FPBMs prepared by RIGC are shown in Table 6. About 99% of the published articles are multi-authored, indicating the presence of collaboration among several research teams locally or internationally. The top 10 countries with 210 publications occupy 62.86% of the total fund records in FPBMs prepared by RIGC. The % of the total fund was calculated from the publications from 1985 to 2021 by dividing the number of funds of the country by the total funds of all countries multiplied by 100. For instance, the USA (37 TP, 15.7% of fund) and Japan (33 TP, 14.9% of fund) are leading the list in terms of citations and the number of publications. For Japan, this can be attributed to the pioneering studies by leading researchers such as T. Okano and the effective governmental plans on research funds for research institutes (e.g., to Tokyo Women Medical University, Center Excellence Century). As for the USA, such a high funding percentage reflects the presence of international collaboration networks, activities, and facilities driven by the collaboration with US institutions and companies. Concerning the ratio between TP and the country’s population, Portugal, with its small population, portrays a typical example of high research intensity in FPBMs prepared by RIGC.

**Figure 7:**
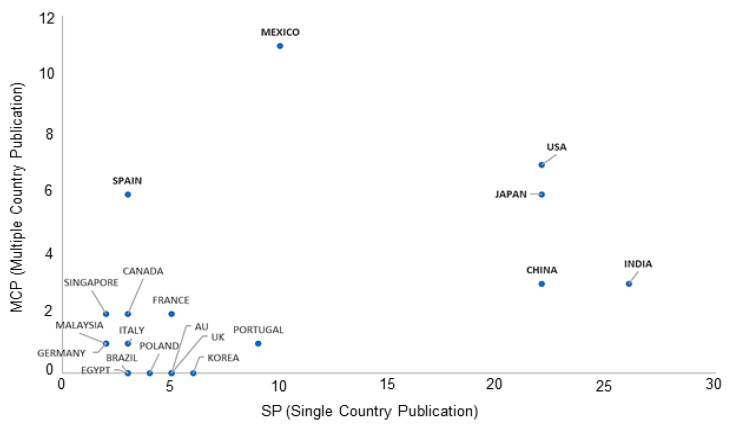
Corresponding author–country collaboration represented by relationship between multiple country and single country publications.

Several national and international research funds and grant programmes have been launched in many countries to focus on promoting interdisciplinary research and the approach towards international collaboration has improved both the quality of the research and inter-profession communication. The corresponding author’s country and the presence of authors from other countries show the preference for international cooperation by the respective corresponding authors. Particularly, the ratio of multiple country publications (MCP) to total publications for most of the top 16 countries is between 10–67%. Mexico (67%) and Spain (52%), which were found to have a strong collaboration as shown in Figure 7. Although the USA and Japan have more MCP than China, India, and the countries in the lower left group, they have a relatively low MCP to TP, standing at 24% and 21%, respectively. Other notable countries in terms of MCP to TP that have a low number of publications are the groups in the lower left corner, with values ranging from 25 to 50%. This indicates a higher preference and importance of the international collaboration for these countries for the research on FPBMs prepared by RIGC. Mapping the collaboration among various countries revealed the presence of 173 collaboration networks among 26 countries, of which Mexico (22), Spain (18), and the USA (18) have the highest numbers of collaboration networks.

### 3.9. Keywords Analysis

**Figure 8:**
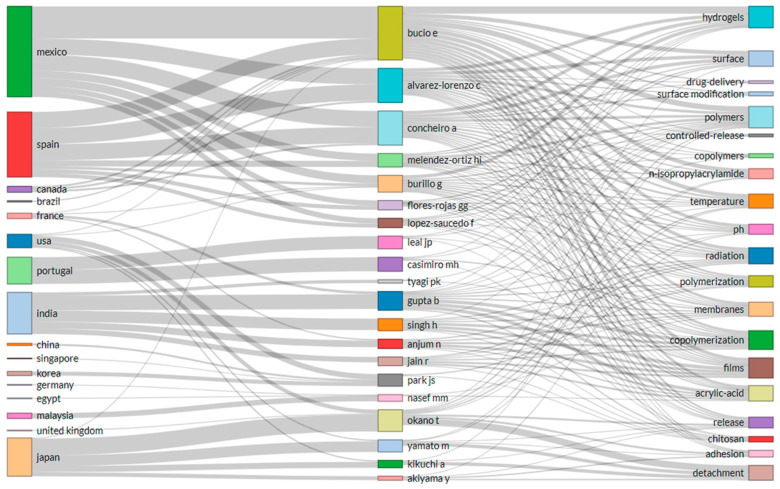
Three-factor plots of the relationship among countries–authors keywords (keywords cloud generated from titles and abstracts) for FPBMs prepared by RIGC literatures. Obtained from that was obtained using Biblioshiny (R-Studio) software.

A more visualised representation of the top researchers, their countries, and specific areas of interest in the field of FPBMs prepared by RIGC is shown in Figure 8. The emphasis of the three-factor plot is placed on the height of each box and the thickness of the connecting lines to the taller boxes both indicate significance, and the thicker the correlation line, the more information or volume of the work can be obtained. From the country viewpoint, both Mexico (7) and Spain (7) have the highest numbers of the authors who eventually prevailed as the top three authors in this research area in terms of author’s affiliations. Surprisingly, all seven authors from Mexico and Spain have cross collaboration involving these two countries. India has five authors in the field of FPBMs prepared by RIGC led by B. Gupta. In terms of affiliations, Emilio Bucio (six) has the highest number of country affiliations followed by G. Burillo (four). The Japanese authors (four) are found to have affiliations with the USA. Authors from Mexico and Spain seem to be focusing on multi-level research as indicated by the diversity in research keywords. E. Bucio, C. Alvarez-Lorenzo, and A. Concheiro focused on 18 of the top 20 keywords, followed by G. Burillo and H. I. Melendez-Ortiz at 13. B. Gupta was found to be focused on 12 keywords, including polymerisation and chitosan, among others. Hydrogels (14), polymers (13), polymerisation (12), copolymerisation (11), chitosan (10), acrylic acid (10), membrane (10), *N*-isopropylacrylamide (nine), and radiation (eight) are the important keywords found in the three-factor plot among county–author keywords.

**Figure 9:**
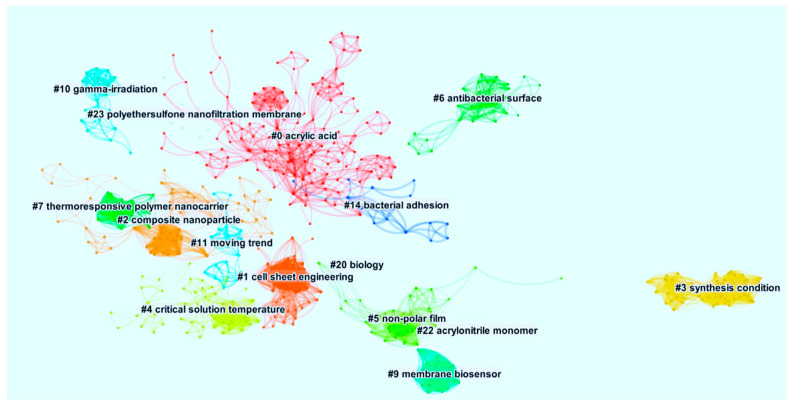
Co-occurrence of clustered keywords network of publications on FPBMs prepared by RIGC. (Obtained by VOSViewer).

Keyword co-occurrence can effectively reflect the hotspots of research areas, allowing exploration of the research trends and providing decision-making directions. The keyword co-occurrence network of publications related to FPBMs prepared by RIGC was constructed from 235 articles. The nodes in the map represent the corresponding keywords and the node size represented the number of publications in the field of RIGC that include the keywords. The link lines between the nodes represent the relationships between the keywords. The 235 articles were mapped to create a thematic concentration for the similar keywords represented by the clusters. Figure 9 shows the co-occurrence of clustered keywords network of publications on FPBMs prepared by RIGC. As can be seen, the co-occurrence of keywords knowledge map revealed keywords with higher centrality and occurrence counts and could be clustered into 15 main sub-clusters, including #0 Acrylic Acid, #1 Cell Sheet Engineering, #2 Composite Nanoparticle, #3 Synthesis Condition, #4 Critical Solution Temperature, #5 Non-Polar Film, #6 Antibacterial Surface, #7 Thermo-responsive, Polymer Nanocarrier, #9 Membrane Biosensor, #10 Gamma-Irradiation, #14 Moving Trend, #14 Bacterial Adhesion, #20 Biology, #22 Acrylonitrile Monomer, and #23 Polyethersulfone Nanofiltration Membrane.

**Figure 10:**
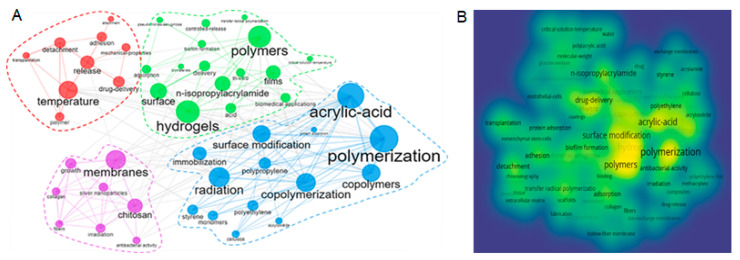
Keywords related to FPBMs obtained by RIGC: (**A**) combined network visualisation and (**B**) density map of co-occurrence. (Obtained from VOSViewer).

Figure 10 presents a combined network visualisation and a density map of co-occurrence of the keywords related to the radiation-grafted FPBMs. Four interrelated groups can be observed. The size of the nodes represents the frequency of the keywords and the thickness of the links between two nodes represents the co-occurrence of the keywords. Although the networks are prepared based on the period 1985–2021, the evolution of these keywords can be found in Figure 10A. Moreover, the map in Figure 10B shows the density of the research keywords. Polymerisation, hydrogels, polymers, acrylic-acid, surface modification, and drug-delivery are among the heavy research areas.

Reference analysis is one of the most common types of analysis in the field of bibliometrics and a clustered network of co-cited references was further performed and could be shown by timeline visualisation. The modularity which is a multiplicative constant representing the number of edges falling within groups divided by the expected number in an equivalent network with edges placed at random. Values close to 1 indicate strong community structure. The network has modularity of 0.885, which suggests that the research on FPBMs prepared by RIGP is clearly defined considering co-citation clusters. The clusters are labelled by index terms selected from the citation frequencies are arranged vertically in descending order of cluster size, and the curves represent the co-citation links between clusters. The nodes with red tree rings or large sizes are references that required more attention because of their high citation number, citation bursts, or both [208].

Further analysis of the co-citation clusters of keywords in the period of 1985–2021 was performed using CiteSpace for the timeline overview prevailed that the duration varied considerably among clusters. After extracting from the landscape of the co-citation network in terms of cluster size, the major clusters with elevated levels of homogeneity are presented in Table 7. The first two columns present the cluster ID and their respective sizes. The “Silhouette” measures in the third column are of greater importance in the clustering of keywords as it indicates the matching between the publications within the cluster and clustering. The closer of the value of “Silhouette” to 1, the higher the homogeneity of the network. In general, these clusters present the main subdomains in FPBMs prepared by RIGP research and are closely related to each other. Some clusters may not be the larger ones, but they are the hottest in this research area. The cluster labelled as “Acrylic Acid” contains 133 publications across a 58-year period from 1962 to 2020. From the timeline, it can be deduced that the initial conceptualisation, development, and expansion of this cluster are going together with the bursts. Surprisingly, the longest surviving cluster in FPBMs prepared by RIGC is the Thermo-responsive Polymer Nanocarrier, which has a span of 18 years and is still active. This clearly shows that this cluster has a long-standing impact on the development of this research area. The cluster of Biology lasted for 16 years in this research area. Other major active clusters include Cell Sheet Engineering, Composite Nanoparticles, Antibacterial Surfaces, and Gamma-irradiation.

### 3.10. Limitation of Study

The restriction of the keywords to radiation grafted functional biopolymers *, OR bioactive surfaces *, OR medical *, OR biotechnological *, OR biomedical *, OR health care * in the titles and abstracts; the search results may not cover all the studies related to FPBMs prepared by RIGC. This is because some authors may not have mentioned the applications, neither in the title nor in the abstract. This is most likely because their studies were still at the preliminary or the fundamental stage. Another account in which the search of keywords may have not been covered is in the studies in which combined techniques, including RIGC and other living polymerisation methods, such as reversible addition fragmentation chain transfer (RAFT) polymerisation, may have been used. This is most likely led to modified names representing the new hybrid techniques such as “radiation induced RAFT polymerization” or “RAFT mediated radiation graft polymerization” that could not be captured. Nevertheless, such studies are not many as compared to the mainstream using conventional RIGC.

## 4. Conclusions and Outlook

The last four decades have witnessed an intense concern in the synthesis of novel radiation-grafted polymers with bioactive surfaces for use in bio-driven applications. The present bibliometric analysis has delivered an overview of FPBMs prepared by RIGC publication trends based on 235 articles selected from the WoS. The study demonstrated that the number of publications related to these materials have been enhanced after 2002, with the lowest and highest numbers of publications being in 1999 and 2012, respectively. The scientometric findings revealed that the applications of FPBMs fall into four main categories, including medical, biomedical, biotechnological, and healthcare applications. Moreover, all applications were dominated by the drug carrier/release materials, which stand at 21.33%, followed by 12.0% for antimicrobial fabrics/films, 11.33% for surgical sutures, 8.67% for scaffolds/tissue engineering, 8% for antifouling membranes, 7.33% for cell culture plates, 6.67% for supports in enzyme immobilisation/release, 6% for catheters, 4.67% for implants, 4.67% for biosensors, 2.67% for packaging films, and 2% for antimicrobial face masks. The Radiation Physics and Chemistry journal is found to be the most dominant journal in publishing articles related to FPBMs prepared by RIGC and applications. Authors such as T. Okano, B. Gupta, H. Singh, H. Feil, and B. Emilio are found to be the most influential researchers in research and collaboration on the preparation and applications of radiation-grafted FPBMs. Countries such as the USA, Japan, and India are the most globally active hubs in research on FPBMs prepared by RIGC, as indicated by the level of international contributions and the sequence of the total funds of 15.7%, 14.9%, and 11.9%, respectively. This coincided with being the leading country in terms of the absolute number of publications and the highest citations.

The drop in publications that took place in the past several years is most likely caused by a combination of factors, such as a lack of funds and less involvement of researchers from universities. This is due to the captivity of irradiation facilities in industries or in radiation research institutes (i.e., facilities are dedicated to certain products/applications), which certainly led to limited access to such external researchers. This is coupled with the competition from other cheaper and well-established technologies of physical immobilisation of bioactive agents to polymer substrates. Nevertheless, several recommendations have been made in recent studies to promote the applications of radiation-grafted FPBMs, including the use of RIGC in the fabrication of more tuneable pH and thermo-responsive polymers for tissue engineering, drug delivery, and medical devices in addition to polymers for implants and antibacterial applications. Finally, it can be concluded that appropriate raising of research funds and establishing systematic collaborative research are required not only to use the RIGC method widely and effectively to design more sustainable, non-toxic, affordable, and timely-needed FPBMs products to advance medical, biomedical, biotechnological, and healthcare fields but also to promote such technology among new generations of young researchers and ultimately help to promote a healthier society in the coming years.

## Figures and Tables

**Table 1:** Classification of FPBMs prepared by RIGC.

Field	Applications	References
Medical	Implants	[60,67,68,69,70,71,72,73,74]
	Catheter	[58,59,75,76,77,78,79,80,81]
	Surgical sutures	[54,82,83,84,85,86,87,88,89,90,91,92,93,94,95,96,97,98]
	Wound dressing hydrogels/patches	[61,99,100,101,102,103,104,105]
Biomedical	Scaffolds for tissue engineering	[56,106,107,108,109,110,111,112,113,114,115,116,117]
	Cell culture plates	[55,118,119,120,121,122,123,124,125,126,127]
	Drug release carrier/delivery	[49,50,52,53,54,83,102,128,129,130,131,132,133,134,135,136,137,138,139,140,141,142,143,144,145,146,147,148,149,150,151,152]
Biotechnological	Biosensors	[50,146,147,148,149,150,151]
	Support for enzyme immobilization/release	[58,153,154,155,156,157,158,159,160,161]
	Antifouling membranes	[66,162,163,164,165,166,167,168,169,170,171,172]
Health care	Antimicrobial fabrics and films	[63,173,174,175,176,177,178,179,180,181,182,183,184,185,186,187,188,189]
	Protective face mask	[62,63,190]
	Packaging films	[64,65,191,192]

**Table 2:** Highly influential journals in relation to published articles on FPBMs prepared by RIGC.

Source	TP (%), 1985–2021	TC	Publisher	Country	IF 2021	Quartile (Q)
Radiation Physics and Chemistry	28 (11.96)	904	Elsevier	UK	2.858	1
Journal of Applied Polymer Science	16 (6.83)	856	Wiley	USA	3.057	2
ACTA Biomaterialia	9 (3.84)	640	Elsevier	Netherlands	10.633	1
Nuclear Instruments and Methods in Physics Research Section B: Beam Interactions with Materials and Atoms	8 (3.40)	395	Elsevier	Netherlands	1.377	2
Progress in Polymer Science	8 (3.40)	5506	Elsevier	UK	31.281	1
Biomaterials	9 (3.01)	2241	Elsevier	UK	12.479	1
Polymer	6 (2.56)	754	Elsevier	Netherlands	4.967	1
Macromolecules	5 (2.13)	378	American Chemical Society	USA	6.057	1
Materials	5 (2.13)	270	MDPI	Switzerland	3.748	2
Langmuir	4 (1.71)	1010	American Chemical Society	USA	4.331	1

Q = Quartile index; TP = Total publications; TC = Total citations; IF = Impact factor.

**Table 3:** Top 10 highly cited review papers on FPBMs prepared by RIGC from 1985 to 2021.

Rank	Title, Ref.	DOI	First Author—Corresponding Author *	Countries’ Contribution	Source, (Q 2021), IF 2021	TC (1985–2021)	C/Y *	Open Access Designation
1	Emerging applications of stimuli-responsive polymer materials, [107]	10.1038/NMAT2614	Stuart, Martien A. Cohen—Igor Luzinov *, Sergiy Minko *	Netherlands, UK, USA	Nature materials, (1), 43.841	4011	364.63	-
2	Hydrogels in biology and medicine: from molecular principles to bionanotechnology, [197]	10.1002/adma.200501612	Peppas, Nicholas A. *—Langer, Robert *	USA	Advanced materials, (1), 32.086	2689	179.26	-
3	Foreign body reaction to biomaterials, [198]	10.1016/j.smim.2007.11.004	Anderson, James M. -Analiz Rodriguez *	USA	Seminars in immunology, (1), 8.856	2648	203.69	Green accepted
4	Stimuli-responsive polymers and their bioconjugates, [29]	10.1016/j.progpolymsci.2004.08.003	Eun Seok Gil—Samuel M.Hudson *	USA	Progress in polymer science (1), 31.281	1976	116.23	-
5	Conducting polymers in biomedical engineering, [199]	10.1016/j.progpolymsci.2007.05.012	Nathalie K.Guimard—Christine E.Schmidt *	USA	Progress in polymer science (1), 31.281	1059	75.64	-
6	Conductive polymers: towards a smart biomaterial for tissue engineering, [200]	10.1016/j.actbio.2014.02.015	Richard Balint—Sarah H.Cartmell *	UK	Acta Biomaterialia, (1), 10.121	860	122.85	Green published, hybrid
7	Polymeric materials with antimicrobial activity, [62]	-	Alexandra Muñoz-Bonilla *—MartaFernández-García *	Spain	Progress in polymer science (1), 31.281	816	90.66	-
8	Thermoresponsive Polymers for Biomedical Applications, [201]	10.3390/polym3031215	Mark A. Ward *	UK	Polymers (1), 4.967	678	67.8	Green submitted, gold
9	Electrochemical sensors based on conducting polymer- polypyrrole, [202]	10.1016/j.electacta.2005.11.052	‪Arunas Ramanaviciu *	Lithuania	Electrochimica Acta (1), 6.901	640	40	Open access
10	Antibacterial surfaces: the quest for a new generation of biomaterials, [203]	10.1016/j.tibtech.2013.01.017	JafarHasan, Elena P. Ivanova *	Australia	Trends in biotechnology (1), 19.53	548	60.88	Open Access

C/Y = Citation per year; TC = Total citations; IF = Impact factor.

**Table 4:** Top 10 highly cited original research articles on FPBMs prepared by RIGC.

Rank	Title/Reference	Application	DOI	First Author—Research Leader *	Countries’ Contribution	Source, (Q 2021), (IF 2021)	TC (1985–2021)	C/Y
1	Effect of comonomer hydrophilicity and ionization on the lower critical solution temperature of N-isopropylacrylamide copolymers, [152]	Controlled drug delivery	10.1021/ma00062a016	H. Feil *	Netherlands	Macromolecules, (1), (5.985)	919	32.82
2	Thermo-responsive polymeric surfaces; control of attachment and detachment of cultured cells, [118]	Cell culture	10.1002/marc.1990.030111109	N Yamada/T. Okano *	Japan	Die makromolekulare chemie, rapid communication, (-), (4.839)	750	35.71
3	Ultrathin conformal devices for precise and continuous thermal characterization of human skin, [204]	Biosensor	doi.org/10.1038/nmat3755	R. Chad Webb/J. A. Rogers *	USA, China, Singapore	Nature materials, (1), (47.656)	689	86.12
4	Permanent, non-leaching antibacterial surfaces—2: How high density cationic surfaces kill bacterial cells, [187]	Antimicrobial surface	10.1016/j.biomaterials.2007.06.012	H. Murata/A. J. Russell *	USA	Biomaterials, (1), (12.479)	492	35.14
5	Synthesis and characterization of pH- and temperature-sensitive poly(methacrylic acid)/poly(N-isopropylacrylamide) interpenetrating polymeric networks, [205]	Controlled drug delivery	10.1021/ma00128a007	C. S. Brazel *	USA	Macromolecules (1), (5.985)	436	16.76
6	Ultrathin poly(N-isopropylacrylamide) grafted layer on polystyrene surfaces for cell adhesion/detachment control, [120]	Cell culture plates	10.1021/la036139f	Y. Akiyama/ T. Okano *	Japan	Langmuir, (1), (4.331)	432	25.41
7	Functional bioengineered corneal epithelial sheet grafts from corneal stem cells expanded ex vivo on a temperature-responsive cell culture surface, [121]	Cell culture plates	10.1097/01.TP.0000110320.45678.30	K. Nishida/ T. Okano *	Japan	Transplantation, (2), (4.74)	424	24.94
8	Covalent attachment of poly(ethylene glycol) to surfaces, critical for reducing bacterial adhesion, [206]	Antifouling surface	10.1021/LA034032M	P. Kingshott *	Denmark	Langmuir, (1), (4.331)	266	14.77
9	A two year in vivo study of polyvinyl alcohol-hydrogel (PVA-H) artificial meniscus, [207]	Knee implant	10.1016/j.biomaterials.2004.08.028	M. Kobayashi *	Japan	Biomaterials, (1), (12.47)	259	16.18
10	Two-Dimensional Manipulation of Cardiac Myocyte Sheets Utilizing Temperature-Responsive Culture Dishes Augments the Pulsatile Amplitude, [126]	Cell culture plates (Cardiac myocyte tissue)	10.1089/107632701300062732	T. Shimizu/ T. Okano *	Japan	Tissue Engineering: Part A (2), (3.845)	186	18.60

C/Y = Citation per year; TC = Total citations; IF = Impact factor.

**Table 5:** Top 10 influential researchers (based on TC) on FPBMs prepared by RIGC.

Rank	Author	TC (1985–2021)	No of Publication	Affiliation	Country
1	T. Okano	4854	20	Tokyo Women Med Univ, Inst Adv Biomed Engn & Sci, TWIns, Shinjuku Ku, Tokyo 1628666, Japan	Japan
2	M. Yamato	2855	11	Tokyo Women Med Univ, Ctr Excellence Century 21, Inst Adv Biomed Engn & Sci, Shinjuku Ku, Tokyo 1628666, Japan	Japan
3	A. Kikuchi	2075	7	Tokyo Women Med Univ, Ctr Excellence Century 21, Inst Adv Biomed Engn & Sci, Shinjuku Ku, Tokyo 1628666, Japan	Japan
4	B. Emilio	592	30	Univ Nacl Autonoma Mexico, Inst Ciencias Nucl, Dept Quim Radiac & Radioquim, Ciudad Univ, Mexico City 04510, DF, Mexico	Mexico
5	B. Gupta	365	12	Indian Inst Technol, Dept Text Technol, New Delhi 110016, India	India
6	A. Concheiro	300	16	Univ Santiago de Compostela, Dept Farm & Tecnol Farmaceut, Santiago De Compostela 15782, Spain	Spain
7	C. Alvarez-Lorenzo	300	16	Univ Santiago de Compostela, Dept Farm & Tecnol Farmaceut, Santiago De Compostela 15782, Spain	Spain
8	G. Burillo	289	8	Univ Nacl Autonoma Mexico, Inst Ciencias Nucl, Dept Quim Radiac & Radioquim, Mexico City 04510, DF, Mexico	Mexico
9	H. Singh	264	10	Indian Inst Technol, Dept Text Technol, New Delhi 110016, India	India
10	H. I. Melendez-Ortiz	82	7	Univ Nacl Autonoma Mexico, Inst Ciencias Nucl, Dept Quim Radiac & Radioquim, Ciudad Univ, Mexico City 04510, DF, Mexico	Mexico

TC = Total citations.

**Table 6:** Most influential countries that provided funds for FPBMs prepared by RIGC in the period of 1985–2021.

Rank	Country	h-Index	All Citations	All Articles	% of Total Fund
1	USA	28	17,558	37	15.7
2	Japan	25	5135	35	14.9
3	India	21	2041	28	11.9
4	China	19	2179	26	11.1
5	Mexico	16	592	30	12.8
6	Spain	12	1217	19	8.1
7	Portugal	9	1458	10	4.3
8	Canada	8	4514	8	3.4
9	Germany	7	4673	9	3.8
10	UK	7	5863	8	3.4

**Table 7:** Temporal properties of major clusters.

Cluster ID	Size	Silhouette	Mean (Year)	Start	End	Duration	Activeness	Theme
0	133	0.955	2004	1962	2020	58	Active	Acrylic Acid
1	68	0.942	1999	1969	2014	45	Active	Cell Sheet Engineering
2	65	0.941	2008	1992	2018	26	Active	Composite Nanoparticle
3	59	1.000	1975	1959	1989	30	Inactive	Synthesis Condition
4	51	0.959	1990	1945	2006	61	Inactive	Critical Solution Temperature
5	47	0.922	1993	1974	2003	29	Inactive	Non-Polar Film
6	35	0.995	2005	1997	2011	14	Active	Antibacterial Surfaces
7	28	0.994	1972	1899	2017	18	Active	Thermo-responsive Polymer Nanocarrier
9	26	0.984	1987	1956	1995	39	Inactive	Membrane Biosensor
10	20	0.990	2010	1969	2020	51	Active	Gamma-Irradiation
11	19	0.997	2001	1991	2006	15	Inactive	Moving Trend
14	16	0.991	2000	1989	2008	19	Inactive	Bacterial Adhesion
20	11	0.994	1999	1989	2005	16	Inactive	Biology
22	11	0.996	1985	1962	1998	36	Inactive	Acrylonitrile Monomer
23	9	0.999	2003	1986	2007	21	Inactive	Polyethersulfone Nanofiltration Membrane

## Data Availability

Data available by corresponding author on request.

## References

[1] Reddy M., Ponnamma D., Choudhary R., Sadasivuni K.K. (2021). A comparative review of natural and synthetic biopolymer composite scaffolds. Polymers.

[2] Liang Y., Li L., Scott R.A., Kiick K.L. (2017). 50th anniversary perspective: Polymeric biomaterials: Diverse functions enabled by advances in macromolecular chemistry. Macromolecules.

[3] Osorio-Delgado M.A., Henao-Tamayo L.J., Velásquez-Cock J.A., Cañas-Gutierrez A.I., Restrepo-Múnera L.M., Gañán-Rojo P.F., Zuluaga-Gallego R.O., Ortiz-Trujillo I.C., Castro-Herazo C.I. (2017). Biomedical applications of polymeric biomaterials. Dyna.

[4] Neděla O., Slepička P., Švorčík V. (2017). Surface modification of polymer substrates for biomedical applications. Materials.

[5] Nasef M.M., Hegazy E.S.A. (2004). Preparation and applications of ion exchange membranes by radiation-induced graft copolymerization of polar monomers onto non-polar films. Prog. Polym. Sci..

[6] Pino-Ramos V.H., Ramos-Ballesteros A., López-Saucedo F., López-Barriguete J.E., Varca G.H., Bucio E. (2016). Radiation grafting for the functionalization and development of smart polymeric materials. Top. Curr. Chem..

[7] Small M., Faglie A., Craig A.J., Pieper M., Fernand Narcisse V.E., Neuenschwander P.F., Chou S.-F. (2018). Nanostructure-enabled and macromolecule-grafted surfaces for biomedical applications. Micromachines.

[8] Ashfaq A., Clochard M.-C., Coqueret X., Dispenza C., Driscoll M.S., Ulański P., Al-Sheikhly M. (2020). Polymerization reactions and modifications of polymers by ionizing radiation. Polymers.

[9] Nasef M.M. (2014). Radiation-grafted membranes for polymer electrolyte fuel cells: Current trends and future directions. Chem. Rev..

[10] Nasef M.M., Gürsel S.A., Karabelli D., Güven O. (2016). Radiation-grafted materials for energy conversion and energy storage applications. Prog. Polym. Sci..

[11] Nasef M.M., Gupta B., Shameli K., Verma C., Ali R.R., Ting T.M. (2021). Engineered Bioactive Polymeric Surfaces by Radiation Induced Graft Copolymerization: Strategies and Applications. Polymers.

[12] Ishigaki I., Sugo T., Senoo K., Okada T., Okamoto J., Machi S. (1982). Graft polymerization of acrylic acid onto polyethylene film by preirradiation method. I. Effects of preirradiation dose, monomer concentration, reaction temperature, and film thickness. J. Appl. Polym. Sci..

[13] Makuuchi K., Cheng S. (2012). Radiation Processing of Polymer Materials and Its Industrial Applications.

[14] Yuan J., Yu C., Peng J., Wang Y., Ma J., Qiu J., Li J., Zhai M. (2013). Facile synthesis of amphoteric ion exchange membrane by radiation grafting of sodium styrene sulfonate and N, N-dimethylaminoethyl methacrylate for vanadium redox flow battery. J. Polym. Sci. Part A Polym. Chem..

[15] Rajabalizadeh Mojarrad N., Sadeghi S., Yarar Kaplan B.m., Güler E., Alkan Gürsel S. (2019). Metal-Salt Enhanced Grafting of Vinylpyridine and Vinylimidazole Monomer Combinations in Radiation Grafted Membranes for High-Temperature PEM Fuel Cells. ACS Appl. Energy Mater..

[16] Lim K.L., Wong C.Y., Wong W.Y., Loh K.S., Selambakkannu S., Othman N.A.F., Yang H. (2021). Radiation-Grafted Anion-Exchange Membrane for Fuel Cell and Electrolyzer Applications: A Mini Review. Membranes.

[17] Hoshina H., Kasai N., Amada H., Takahashi M., Tanaka K., Seko N. (2014). Recovery of scandium from hot spring water with graft adsorbent containing phosphoric groups. J. Ion Exch..

[18] Hanh T.T., Huy H.T., Hien N.Q. (2015). Pre-irradiation grafting of acrylonitrile onto chitin for adsorption of arsenic in water. Radiat. Phys. Chem..

[19] Gao Q., Hu J., Li R., Xing Z., Xu L., Wang M., Guo X., Wu G. (2016). Radiation synthesis of a new amidoximated UHMWPE fibrous adsorbent with high adsorption selectivity for uranium over vanadium in simulated seawater. Radiat. Phys. Chem..

[20] Sawada S.-i., Maekawa Y. (2021). Radiation-Induced Asymmetric Grafting of Different Monomers into Base Films to Prepare Novel Bipolar Membranes. Molecules.

[21] Ishihara R., Asai S., Saito K. (2020). Recent Progress in Charged Polymer Chains Grafted by Radiation-Induced Graft Polymerization; Adsorption of Proteins and Immobilization of Inorganic Precipitates. Quantum Beam Sci..

[22] Jaganathan S.K., Balaji A., Vellayappan M.V., Subramanian A.P., John A.A., Asokan M.K., Supriyanto E. (2015). Radiation-induced surface modification of polymers for biomaterial application. J. Mater. Sci..

[23] Chapiro A. (1962). Radiation Chemistry of Polymer System.

[24] Charlsby A. (1960). Atomic Radiation and Polymers.

[25] Nasef M.M., Güven O. (2012). Radiation-grafted copolymers for separation and purification purposes: Status, challenges and future directions. Prog. Polym. Sci..

[26] Dong Z., Wang Y., Wen D., Peng J., Zhao L., Zhai M. (2021). Recent Progress in Environmental Applications of Functional Adsorbent Prepared by Radiation techniques: A review. J. Hazard. Mater..

[27] Gubler L., Scherer G.G. (2010). Trends for fuel cell membrane development. Desalination.

[28] Hoffman A.S., Stayton P.S., Bulmus V., Chen G., Chen J., Cheung C., Chilkoti A., Ding Z., Dong L., Fong R. (2000). Really smart bioconjugates of smart polymers and receptor proteins. J. Biomed. Mater. Res..

[29] Gil E.S., Hudson S.M. (2004). Stimuli-reponsive polymers and their bioconjugates. Prog. Polym. Sci..

[30] Hoffman A.S. (1991). Conventional and environmentally-sensitive hydrogels for medical and industrial uses: A review paper. Polymer Gels.

[31] Carenza M. (1992). Recent achievements in the use of radiation polymerization and grafting for biomedical applications. Int. J. Radiat. Appl. Instrum. Part C Radiat. Phys. Chem..

[32] Ulanski P., Janik I., Kadlubowski S., Kozicki M., Kujawa P., Pietrzak M., Stasica P., Rosiak J.M. (2002). Polymeric biomaterials synthesized by radiation techniques–current studies at IARC, Poland. Polym. Adv. Technol..

[33] Rosiak J., Janik I., Kadlubowski S., Kozicki M., Kujawa P., Stasica P., Ulanski P. (2003). Nano-, micro-and macroscopic hydrogels synthesized by radiation technique. Nucl. Instrum. Methods Phys. Res. Sect. B Beam Interact. Mater. At..

[34] Xu X., Liu Y., Fu W., Yao M., Ding Z., Xuan J., Li D., Wang S., Xia Y., Cao M. (2020). Poly (N-isopropylacrylamide)-based thermoresponsive composite hydrogels for biomedical applications. Polymers.

[35] Wach R.A., Rosiak J.M., Ulański P. Polysaccharides hydrogel-radiation induced formation and medical applications. Proceedings of the 26th Biomaterials in Medicine and Veterinary Medicine Conference.

[36] Yang J., Yamato M., Kohno C., Nishimoto A., Sekine H., Fukai F., Okano T. (2005). Cell sheet engineering: Recreating tissues without biodegradable scaffolds. Biomaterials.

[37] Yamato M., Akiyama Y., Kobayashi J., Yang J., Kikuchi A., Okano T. (2007). Temperature-responsive cell culture surfaces for regenerative medicine with cell sheet engineering. Prog. Polym. Sci..

[38] Teles M.N.O., Santos B.L.P., Silva D.P., Teixeira J.A., Ruzene D.S. (2020). A bibliometric description of lignin applicability for the removal of chemical pollutants in effluents. Water Air Soil Pollut..

[39] Sweileh W.M. (2021). Bibliometric analysis of peer-reviewed literature on antimicrobial stewardship from 1990 to 2019. Glob. Health.

[40] Almeida F.L.C., Castro M.P.J., Travália B.M., Forte M.B.S. (2021). Trends in lipase immobilization: Bibliometric review and patent analysis. Process Biochem..

[41] Salgado-Cruz M.d.l.P., Salgado-Cruz J., García-Hernández A.B., Calderón-Domínguez G., Gómez-Viquez H., Oliver-Espinoza R., Fernández-Martínez M.C., Yáñez-Fernández J. (2021). Chitosan as a Coating for Biocontrol in Postharvest Products: A Bibliometric Review. Membranes.

[42] Ghule B., Laad M. (2020). A Bibliometric Survey on Polymer Composites in Energy Storage Applications. Libr. Philos. Pract..

[43] Khan A.S., Ur Rehman S., AlMaimouni Y.K., Ahmad S., Khan M., Ashiq M. (2020). Bibliometric Analysis of Literature Published on Antibacterial Dental Adhesive from 1996–2020. Polymers.

[44] Simmons P., McElroy T., Allen A.R. (2020). A bibliometric review of artificial extracellular matrices based on tissue engineering technology literature: 1990 through 2019. Materials.

[45] Abejón R., Pérez-Acebo H., Garea A. (2017). A bibliometric analysis of research on supported ionic liquid membranes during the 1995–2015 period: Study of the main applications and trending topics. Membranes.

[46] Gonçalves M.C.P., Kieckbusch T.G., Perna R.F., Fujimoto J.T., Morales S.A.V., Romanelli J.P. (2019). Trends on enzyme immobilization researches based on bibliometric analysis. Process Biochem..

[47] Van Eck N.J., Waltman L. (2014). Visualizing bibliometric networks. Measuring Scholarly Impact.

[48] Aria M., Cuccurullo C. (2017). bibliometrix: An R-tool for comprehensive science mapping analysis. J. Informetr..

[49] Jeong S.I., Park S.-C., Park S.-J., Kim E.-J., Heo H., Park J.-S., Gwon H.-J., Lim Y.-M., Jang M.-K. (2018). One-step synthesis of gene carrier via gamma irradiation and its application in tumor gene therapy. Int. J. Nanomed..

[50] Turmanova S., Trifonov A., Kalaijiev O., Kostov G. (1997). Radiation grafting of acrylic acid onto polytetrafluoroethylene films for glucose oxidase immobilization and its application in membrane biosensor. J. Membr. Sci..

[51] Pino-Ramos V., Meléndez-Ortiz H., Ramos-Ballesteros A., Bucio E. (2018). Radiation Grafting of Biopolymers and Synthetic Polymers. Biopolymer Grafting: Applications.

[52] Alvarez-Lorenzo C., Bucio E., Burillo G., Concheiro A. (2010). Medical devices modified at the surface by γ-ray grafting for drug loading and delivery. Expert Opin. Drug Deliv..

[53] Singh B., Kumar A. (2018). Radiation-induced graft copolymerization of N-vinyl imidazole onto moringa gum polysaccharide for making hydrogels for biomedical applications. Int. J. Biol. Macromol..

[54] García-Vargas M., González-Chomón C., Magariños B., Concheiro A., Alvarez-Lorenzo C., Bucio E. (2014). Acrylic polymer-grafted polypropylene sutures for covalent immobilization or reversible adsorption of vancomycin. Int. J. Pharm..

[55] Tang Z., Akiyama Y., Okano T. (2014). Recent development of temperature-responsive cell culture surface using poly (N-isopropylacrylamide). J. Polym. Sci. Part B Polym. Phys..

[56] Pasparakis G., Tsitsilianis C. (2020). LCST polymers: Thermoresponsive nanostructured assemblies towards bioapplications. Polymer.

[57] Jabbari E., Nozari S. (2000). Swelling behavior of acrylic acid hydrogels prepared by γ-radiation crosslinking of polyacrylic acid in aqueous solution. Eur. Polym. J..

[58] Costoya A., Becerra L.E.V., Meléndez-Ortiz H.I., Díaz-Gómez L., Mayer C., Otero A., Concheiro A., Bucio E., Alvarez-Lorenzo C. (2019). Immobilization of antimicrobial and anti-quorum sensing enzymes onto GMA-grafted poly (vinyl chloride) catheters. Int. J. Pharm..

[59] Zuñiga-Zamorano I., Meléndez-Ortiz H.I., Costoya A., Alvarez-Lorenzo C., Concheiro A., Bucio E. (2018). Poly (vinyl chloride) catheters modified with pH-responsive poly (methacrylic acid) with affinity for antimicrobial agents. Radiat. Phys. Chem..

[60] Hidzir N.M., Radzali N.A.M., Rahman I.A., Shamsudin S.A. (2020). Gamma irradiation-induced grafting of 2-hydroxyethyl methacrylate (HEMA) onto ePTFE for implant applications. Nucl. Eng. Technol..

[61] Hiriart-Ramírez E., Contreras-García A., Garcia-Fernandez M.J., Concheiro A., Alvarez-Lorenzo C., Bucio E. (2012). Radiation grafting of glycidyl methacrylate onto cotton gauzes for functionalization with cyclodextrins and elution of antimicrobial agents. Cellulose.

[62] Muñoz-Bonilla A., Fernández-García M. (2012). Polymeric materials with antimicrobial activity. Prog. Polym. Sci..

[63] Aoki S., Fujiwara K., Sugo T., Suzuki K. (2013). Antimicrobial fabric adsorbed iodine produced by radiation-induced graft polymerization. Radiat. Phys. Chem..

[64] Riquet A.-M., Delattre J., Vitrac O., Guinault A. (2013). Design of modified plastic surfaces for antimicrobial applications: Impact of ionizing radiation on the physical and mechanical properties of polypropylene. Radiat. Phys. Chem..

[65] Ping X., Wang M., Xuewu G. (2011). Surface modification of poly (ethylene terephthalate)(PET) film by gamma-ray induced grafting of poly (acrylic acid) and its application in antibacterial hybrid film. Radiat. Phys. Chem..

[66] Lim S.J., Shin I.H. (2020). Graft copolymerization of GMA and EDMA on PVDF to hydrophilic surface modification by electron beam irradiation. Nucl. Eng. Technol..

[67] Stasica P., Rosiak J., Ciach M., Radek M. (2000). Approach to construct hydrogel intervertebral disc implants—Experimental and numerical investigations. Eng. Biomater..

[68] Yoshii F., Makuuchi K., Sudradjat A., Darwis D., Razzak M. (1992). Heat stability of radiation crosslinked poly (vinyl alcohol) hydrogel. Ika Kikaigaku.

[69] Darwis D., Stasica P., Razzak M.T., Rosiak J.M. (2002). Characterization of poly (vinyl alcohol) hydrogel for prosthetic intervertebral disc nucleus. Radiat. Phys. Chem..

[70] Lee S.-H., An S.-J., Lim Y.-M., Huh J.-B. (2017). The efficacy of electron beam irradiated bacterial cellulose membranes as compared with collagen membranes on guided bone regeneration in peri-implant bone defects. Materials.

[71] Hidzir N.M., Hill D.J., Martin D., Grøndahl L. (2012). Radiation-induced grafting of acrylic acid onto expanded poly (tetrafluoroethylene) membranes. Polymer.

[72] Magaña H., Becerra C.D., Serrano-Medina A., Palomino K., Palomino-Vizcaíno G., Olivas-Sarabia A., Bucio E., Cornejo-Bravo J.M. (2020). Radiation Grafting of a Polymeric Prodrug onto Silicone Rubber for Potential Medical/Surgical Procedures. Polymers.

[73] Kyomoto M., Moro T., Saiga K., Hashimoto M., Ito H., Kawaguchi H., Takatori Y., Ishihara K. (2012). Biomimetic hydration lubrication with various polyelectrolyte layers on cross-linked polyethylene orthopedic bearing materials. Biomaterials.

[74] Moro T., Kawaguchi H., Ishihara K., Kyomoto M., Karita T., Ito H., Nakamura K., Takatori Y. (2009). Wear resistance of artificial hip joints with poly (2-methacryloyloxyethyl phosphorylcholine) grafted polyethylene: Comparisons with the effect of polyethylene cross-linking and ceramic femoral heads. Biomaterials.

[75] Meléndez-Ortiz H.I., Alvarez-Lorenzo C., Concheiro A., Jimenez-Paez V.M., Bucio E. (2016). Modification of medical grade PVC with N-vinylimidazole to obtain bactericidal surface. Radiat. Phys. Chem..

[76] Meléndez-Ortiz H.I., Alvarez-Lorenzo C., Burillo G., Magariños B., Concheiro A., Bucio E. (2015). Radiation-grafting of N-vinylimidazole onto silicone rubber for antimicrobial properties. Radiat. Phys. Chem..

[77] Valencia-Mora R.A., Zavala-Lagunes E., Bucio E. (2016). Grafting of thermo-sensitive N-vinylcaprolactam onto silicone rubber through the direct radiation method. Radiat. Phys. Chem..

[78] Razzak M.T., Otsuhata K., Tabata Y., Ohashi F., Takeuchi A. (1989). Modification of natural rubber tubes for biomaterials. II. Radiation-induced grafting of N, N-dimethylaminoethylacrylate (DMAEA) onto natural rubber (NR) tubes. J. Appl. Polym. Sci..

[79] Razzak M.T., Otsuhata K., Tabata Y., Ohashi F., Takeuchi A. (1988). Modification of natural rubber tubes for biomaterials I. Radiation-induced grafting of N, N-dimethyl acrylamide onto natural rubber tubes. J. Appl. Polym. Sci..

[80] Nowatzki P.J., Koepsel R.R., Stoodley P., Min K., Harper A., Murata H., Donfack J., Hortelano E.R., Ehrlich G.D., Russell A.J. (2012). Salicylic acid-releasing polyurethane acrylate polymers as anti-biofilm urological catheter coatings. Acta Biomater..

[81] Hosny A.E.-D.M., Farrag H.A., Helmy O.M., Hagras S.A., El-Hag Ali A. (2020). In-vitro evaluation of antibacterial and antibiofilm efficiency of radiation-modified polyurethane–ZnO nanocomposite to be used as a self-disinfecting catheter. J. Radiat. Res. Appl. Sci..

[82] Mukherjee A., Gupta B. (1985). Radiation-induced graft copolymerization of methacrylic acid onto polypropylene fibers. I. Effect of synthesis conditions. J. Appl. Polym. Sci..

[83] Singh H., Tyagi P. (1989). Radiation induced grafting of methacrylic acid onto silk for the immobilization of antimicrobial drug for sustained delivery. Die Angew. Makromol. Chem. Appl. Macromol. Chem. Phys..

[84] Tyagi P., Gupta B., Singh H. (1993). Radiation-induced grafting of 2-hydroxyethyl methacrylate onto polypropylene for biomedical applications. II. Evaluation as antimicrobial suture. J. Macromol. Sci. Part A Pure Appl. Chem..

[85] Plessier C., Gupta B., Chapiro A. (1998). Modification of polypropylene fiber by radiation-induced graft copolymerization of acrylonitrile monomer. J. Appl. Polym. Sci..

[86] Gupta B., Anjum N., Gulrez S., Singh H. (2007). Development of antimicrobial polypropylene sutures by graft copolymerization. II. Evaluation of physical properties, drug release, and antimicrobial activity. J. Appl. Polym. Sci..

[87] Yuan F., Wei J., Tang E.-Q., Zhao K.-Y., Xue Y. (2009). Synthesis and Modification of Polypropylene by Radiation-induced Grafting. Int. J. Chem..

[88] López-Saucedo F., Flores-Rojas G.G., López-Saucedo J., Magariños B., Alvarez-Lorenzo C., Concheiro A., Bucio E. (2018). Antimicrobial silver-loaded polypropylene sutures modified by radiation-grafting. Eur. Polym. J..

[89] López-Saucedo F., Alvarez-Lorenzo C., Concheiro A., Bucio E. (2017). Radiation-grafting of vinyl monomers separately onto polypropylene monofilament sutures. Radiat. Phys. Chem..

[90] Marisol Arteaga-Luna M., Hugo Pino-Ramos V., Magaña H., Bucio E. (2018). Polymeric pro-drug sutures for potential local release of salicylic acid. Int. J. Polym. Mater. Polym. Biomater..

[91] López-Saucedo F., Flores-Rojas G., Bucio E., Alvarez-Lorenzo C., Concheiro A., González-Antonio O. (2017). Achieving antimicrobial activity through poly (N-methylvinylimidazolium) iodide brushes on binary-grafted polypropylene suture threads. MRS Communications.

[92] Tummalapalli M., Anjum S., Kumari S., Gupta B. (2016). Antimicrobial surgical sutures: Recent developments and strategies. Polym. Rev..

[93] Buchenska J., Slomkowski S., Tazbir J., Sobolewska E. (2003). Antibacterial poly (ethylene terephthalate) yarn containing cephalosporin type antibiotic. Fibres Text. East. Eur..

[94] Anjum N., Gulrez S., Singh H., Gupta B. (2006). Development of antimicrobial polypropylene sutures by graft polymerization. I. Influence of grafting conditions and characterization. J. Appl. Polym. Sci..

[95] Gupta B., Jain R., Anjum N., Singh H. (2006). Preirradiation grafting of acrylonitrile onto polypropylene monofilament for biomedical applications: I. Influence of synthesis conditions. Radiat. Phys. Chem..

[96] Gupta B., Jain R., Singh H. (2008). Preparation of antimicrobial sutures by preirradiation grafting onto polypropylene monofilament. Polym. Adv. Technol..

[97] Gupta B., Jain R., Anjum N., Singh H. (2004). Preparation of antimicrobial sutures by preirradiation grafting of acrylonitrile onto polypropylene monofilament. III. Hydrolysis of the grafted suture. J. Appl. Polym. Sci..

[98] Jain R., Gupta B., Anjum N., Revagade N., Singh H. (2004). Preparation of antimicrobial sutures by preirradiation grafting of acrylonitrile onto polypropylene monofilament. II. mechanical, physical, and thermal characteristics. J. Appl. Polym. Sci..

[99] Wu M., Bao B., Yoshii F., Makuuchi K. (2001). Irradiation of crosslinked, poly (vinyl alcohol) blended hydrogel for wound dressing. J. Radioanal. Nucl. Chem..

[100] Zhao L., Mitomo H., Zhai M., Yoshii F., Nagasawa N., Kume T. (2003). Synthesis of antibacterial PVA/CM-chitosan blend hydrogels with electron beam irradiation. Carbohydr. Polym..

[101] Yang X., Liu Q., Chen X., Yu F., Zhu Z. (2008). Investigation of PVA/ws-chitosan hydrogels prepared by combined γ-irradiation and freeze-thawing. Carbohydr. Polym..

[102] Abou Taleb M.F., Ismail S.A., El-Kelesh N.A. (2008). Radiation synthesis and characterization of polyvinyl alcohol/methacrylic acid–gelatin hydrogel for vitro drug delivery. J. Macromol. Sci. Part A.

[103] Kaur I., Bhati P., Sharma S. (2014). Radiation induced synthesis of (gelatin-co-PVA)-g-poly (AAc) copolymer as wound dressing material. Adv. Mater. Res..

[104] Razzak M.T., Darwis D. (2001). Irradiation of polyvinyl alcohol and polyvinyl pyrrolidone blended hydrogel for wound dressing. Radiat. Phys. Chem..

[105] Casimiro M., Gil M., Leal J. (2010). Suitability of gamma irradiated chitosan based membranes as matrix in drug release system. Int. J. Pharm..

[106] Cabane E., Zhang X., Langowska K., Palivan C.G., Meier W. (2012). Stimuli-responsive polymers and their applications in nanomedicine. Biointerphases.

[107] Stuart M.A.C., Huck W.T., Genzer J., Müller M., Ober C., Stamm M., Sukhorukov G.B., Szleifer I., Tsukruk V.V., Urban M. (2010). Emerging applications of stimuli-responsive polymer materials. Nat. Mater..

[108] Meléndez-Ortiz I.H., Bucio E. (2009). Stimuli-sensitive behaviour of binary graft Co-polymers (PP-g-DMAEMA)-g-NIPAAm and (PP-g-4VP)-g-NIPAAm in acidic and basic medium. Des. Monomers Polym..

[109] Luk J.Z., Cooper-White J., Rintoul L., Taran E., Grøndahl L. (2013). Functionalised polycaprolactone films and 3D scaffolds via gamma irradiation-induced grafting. J. Mater. Chem. B.

[110] Rahman M., Sreearunothai P., Opaprakasit P. (2017). Development and Characterization of Photoinduced Acrylamide-Grafted Polylactide Films for Biomedical Applications. Int. J. Polym. Sci..

[111] Casimiro M.H., Gomes S.R., Rodrigues G., Leal J.P., Ferreira L.M. (2018). Chitosan/Poly (vinylpyrrolidone) matrices obtained by gamma-irradiation for skin scaffolds: Characterization and preliminary cell response studies. Materials.

[112] Casimiro M.H., Lancastre J.J., Rodrigues A.P., Gomes S.R., Rodrigues G., Ferreira L.M. (2017). Chitosan-Based matrices prepared by gamma irradiation for tissue regeneration: Structural properties vs. preparation method. Applications of Radiation Chemistry in the Fields of Industry, Biotechnology and Environment.

[113] Tang Z., Akiyama Y., Okano T. (2012). Temperature-responsive polymer modified surface for cell sheet engineering. Polymers.

[114] Elloumi-Hannachi I., Yamato M., Okano T. (2010). Cell sheet engineering: A unique nanotechnology for scaffold-free tissue reconstruction with clinical applications in regenerative medicine. J. Intern. Med..

[115] Takahashi H., Nakayama M., Yamato M., Okano T. (2010). Controlled chain length and graft density of thermoresponsive polymer brushes for optimizing cell sheet harvest. Biomacromolecules.

[116] Haraguchi Y., Shimizu T., Yamato M., Okano T. (2012). Scaffold-free tissue engineering using cell sheet technology. RSC Adv..

[117] Riquet A., Rohman G., Guinault A., Demilly M. (2011). Surface modification of polypropylene by radiation grafting of hydrophilic monomers: Physicochemical properties. Surf. Eng..

[118] Yamada N., Okano T., Sakai H., Karikusa F., Sawasaki Y., Sakurai Y. (1990). Thermo-responsive polymeric surfaces; control of attachment and detachment of cultured cells. Die Makromol. Chem. Rapid Commun..

[119] Shimizu T., Yamato M., Kikuchi A., Okano T. (2003). Cell sheet engineering for myocardial tissue reconstruction. Biomaterials.

[120] Akiyama Y., Kikuchi A., Yamato M., Okano T. (2004). Ultrathin poly (N-isopropylacrylamide) grafted layer on polystyrene surfaces for cell adhesion/detachment control. Langmuir.

[121] Nishida K., Yamato M., Hayashida Y., Watanabe K., Maeda N., Watanabe H., Yamamoto K., Nagai S., Kikuchi A., Tano Y. (2004). Functional bioengineered corneal epithelial sheet grafts from corneal stem cells expanded ex vivo on a temperature-responsive cell culture surface. Transplantation.

[122] Fukumori K., Akiyama Y., Yamato M., Kobayashi J., Sakai K., Okano T. (2009). Temperature-responsive glass coverslips with an ultrathin poly (N-isopropylacrylamide) layer. Acta Biomater..

[123] Akiyama Y., Yamato M., Okano T. (2013). Preparation of poly (N-isopropylacrylamide) grafted polydimethylsiloxane by using electron beam irradiation. J. Robot. Mechatron..

[124] Kumar P.A., Sreenivasan K., Kumary T. (2007). Alternate method for grafting thermoresponsive polymer for transferring in vitro cell sheet structures. J. Appl. Polym. Sci..

[125] von Recum H., Okano T., Kim S.W. (1998). Growth factor release from thermally reversible tissue culture substrates. J. Control. Release.

[126] Shimizu T., Yamato M., Kikuchi A., Okano T. (2001). Two-dimensional manipulation of cardiac myocyte sheets utilizing temperature-responsive culture dishes augments the pulsatile amplitude. Tissue Eng..

[127] Kobayashi J., Okano T. (2010). Fabrication of a thermoresponsive cell culture dish: A key technology for cell sheet tissue engineering. Sci. Technol. Adv. Mater..

[128] Muñoz-Muñoz F., Ruiz J.-C., Alvarez-Lorenzo C., Concheiro A., Bucio E. (2009). Novel interpenetrating smart polymer networks grafted onto polypropylene by gamma radiation for loading and delivery of vancomycin. Eur. Polym. J..

[129] Sponchioni M., Palmiero U.C., Moscatelli D. (2019). Thermo-responsive polymers: Applications of smart materials in drug delivery and tissue engineering. Mater. Sci. Eng. C.

[130] Gandhi A., Paul A., Sen S.O., Sen K.K. (2015). Studies on thermoresponsive polymers: Phase behaviour, drug delivery and biomedical applications. Asian J. Pharm. Sci..

[131] Demirdirek B., Uhrich K.E. (2017). Novel salicylic acid-based chemically crosslinked pH-sensitive hydrogels as potential drug delivery systems. Int. J. Pharm..

[132] Vázquez-González B., Meléndez-Ortiz H.I., Díaz-Gómez L., Alvarez-Lorenzo C., Concheiro A., Bucio E. (2014). Silicone rubber modified with methacrylic acid to host antiseptic drugs. Macromol. Mater. Eng..

[133] Kayal S. (2021). Thermoresponsive magnetic/polymer composite nanoparticles for biomedical applications. Mater. Today Proc..

[134] Mutalik S., Suthar N.A., Managuli R.S., Shetty P.K., Avadhani K., Kalthur G., Kulkarni R.V., Thomas R. (2016). Development and performance evaluation of novel nanoparticles of a grafted copolymer loaded with curcumin. Int. J. Biol. Macromol..

[135] Magaña H., Palomino K., Cornejo-Bravo J.M., Alvarez-Lorenzo C., Concheiro A., Bucio E. (2015). Radiation-grafting of acrylamide onto silicone rubber films for diclofenac delivery. Radiat. Phys. Chem..

[136] Melendez-Ortiz H.I., Díaz-Rodríguez P., Alvarez-Lorenzo C., Concheiro A., Bucio E. (2014). Binary graft modification of polypropylene for anti-inflammatory drug–device combo products. J. Pharm. Sci..

[137] De Oliveira M., Parra D., Amato V., Lugão A. (2013). Hydrogel membranes of PVAl/clay by gamma radiation. Radiat. Phys. Chem..

[138] Gagliardi M. (2012). In vitro haematic proteins adsorption and cytocompatibility study on acrylic copolymer to realise coatings for drug-eluting stents. Mater. Sci. Eng. C.

[139] Burillo G., Bucio E., Arenas E., Lopez G.P. (2007). Temperature and pH-sensitive swelling behavior of binary DMAEMA/4VP grafts on poly (propylene) films. Macromol. Mater. Eng..

[140] Bucio E., Burillo G. (2007). Radiation grafting of pH and thermosensitive N-isopropylacrylamide and acrylic acid onto PTFE films by two-steps process. Radiat. Phys. Chem..

[141] Stoica-Guzun A., Stroescu M., Tache F., Zaharescu T., Grosu E. (2007). Effect of electron beam irradiation on bacterial cellulose membranes used as transdermal drug delivery systems. Nucl. Instrum. Methods Phys. Res. Sect. B Beam Interact. Mater. At..

[142] Casimiro M., Gil M., Leal J. (2007). Drug release assays from new chitosan/pHEMA membranes obtained by gamma irradiation. Nucl. Instrum. Methods Phys. Res. Sect. B Beam Interact. Mater. At..

[143] Ramírez-Fuentes Y.S., Bucio E., Burillo G. (2007). Radiation-induced grafting of N-isopropylacrylamide and acrylic acid onto polypropylene films by two step method. Nucl. Instrum. Methods Phys. Res. Sect. B Beam Interact. Mater. At..

[144] Ikram S., Kumari M., Gupta B. (2011). Thermosensitive membranes by radiation-induced graft polymerization of N-isopropyl acrylamide/acrylic acid on polypropylene nonwoven fabric. Radiat. Phys. Chem..

[145] Contreras-García A., Alvarez-Lorenzo C., Taboada C., Concheiro A., Bucio E. (2011). Stimuli–responsive networks grafted onto polypropylene for the sustained delivery of NSAIDs. Acta Biomater..

[146] Piao M.-H., Yang D.-S., Yoon K.-R., Lee S.-H., Choi S.-H. (2009). Development of an electrogenerated chemiluminescence biosensor using carboxylic acid-functionalized MWCNT and Au nanoparticles. Sensors.

[147] Yang J.-H., Lee J.-C., Choi S.-H. (2009). Tyrosinase-immobilized biosensor based on the functionalized hydroxyl group-MWNT and detection of phenolic compounds in red wines. J. Sens..

[148] Yang D.-S., Jung D.-J., Choi S.-H. (2010). One-step functionalization of multi-walled carbon nanotubes by radiation-induced graft polymerization and their application as enzyme-free biosensors. Radiat. Phys. Chem..

[149] Kim S.-K., Kwen H.-D., Choi S.-H. (2011). Fabrication of a microbial biosensor based on QD-MWNT supports by a one-step radiation reaction and detection of phenolic compounds in red wines. Sensors.

[150] Kim K.-I., Kang H.-Y., Lee J.-C., Choi S.-H. (2009). Fabrication of a multi-walled nanotube (MWNT) ionic liquid electrode and its application for sensing phenolics in red wines. Sensors.

[151] Lee Y.-J., Chung D.-J., Oh S.-H., Choi S.-H. (2012). Introduction of bifunctional group onto MWNT by radiation-induced graft polymerization and its use as biosensor-supporting materials. J. Nanomater..

[152] Feil H., Bae Y.H., Feijen J., Kim S.W. (1993). Effect of comonomer hydrophilicity and ionization on the lower critical solution temperature of N-isopropylacrylamide copolymers. Macromolecules.

[153] Pino-Ramos V.H., Flores-Rojas G.G., Alvarez-Lorenzo C., Concheiro A., Bucio E. (2018). Graft copolymerization by ionization radiation, characterization, and enzymatic activity of temperature-responsive SR-g-PNVCL loaded with lysozyme. React. Funct. Polym..

[154] Abd Halin N.I., Al-Khatib M.F.R., Salleh H.M., Nasef M.M. (2019). Preparation and Candida rugosa Lipase Immobilization on Nylon-6 Grafted and Aminated (Polyvinyl Benzyl Chloride) Microfibers. Bull. Chem. React. Eng. Catal..

[155] Alkhatib M., Bahrudin N.A., SALLEH H.M., Nasef M.M., Ting T.M. (2019). Lipase immobilization on fibers grafted with polyglycidyl methachrylate. IIUM Eng. J..

[156] Kamal H., Sabry G.M., Lotfy S., Abdallah N.M., Rosiak J., Hegazy E.s.A. (2007). Immobilization of glucoamylase on polypropylene fibers modified by radiation induced graft copolymerization. J. Macromol. Sci. Part A Pure Appl. Chem..

[157] Garnett J., Jankiewicz S., Levot R., Sangster D. (1985). Insolubilisation of biologically active materials with novel radiation graft copolymers. Radiat. Phys. Chem..

[158] Dong L.C., Hoffman A.S. (1986). Thermally reversible hydrogels: III. Immobilization of enzymes for feedback reaction control. J. Control. Release.

[159] Dong L.C., Hoffman A.S. (1986). A new method for immobilization of biomolecules using preirradiation grafting at low temperature. Int. J. Radiat. Appl. Instrum. Part C. Radiat. Phys. Chem..

[160] Hongfei H., Guanghui W., Jilan W. (1988). Immobilization of peroxidase on SPEU film via radiation grafting. Int. J. Radiat. Appl. Instrum. Part C. Radiat. Phys. Chem..

[161] Schmidt M., Prager A., Schönherr N., Gläser R., Schulze A. (2022). Reagent-free immobilization of industrial lipases to develop lipolytic membranes with self-cleaning surfaces. Membranes.

[162] Xu C., Huang W., Zhou Y., Yan D., Chen S., Huang H. (2012). Graft copolymerization of N-vinyl-2-pyrrolidone onto pre-irradiated poly (vinylidene fluoride) powder. Radiat. Phys. Chem..

[163] Qin Q., Hou Z., Lu X., Bian X., Chen L., Shen L., Wang S. (2013). Microfiltration membranes prepared from poly (N-vinyl-2-pyrrolidone) grafted poly (vinylidene fluoride) synthesized by simultaneous irradiation. J. Membr. Sci..

[164] Shen L., Feng S., Li J., Chen J., Li F., Lin H., Yu G. (2017). Surface modification of polyvinylidene fluoride (PVDF) membrane via radiation grafting: Novel mechanisms underlying the interesting enhanced membrane performance. Sci. Rep..

[165] Xi Z.-Y., Xu Y.-Y., Zhu L.-P., Zhu B.-K. (2009). Modification of polytetrafluoroethylene porous membranes by electron beam initiated surface grafting of binary monomers. J. Membr. Sci..

[166] Deng B., Yang X., Xie L., Li J., Hou Z., Yao S., Liang G., Sheng K., Huang Q. (2009). Microfiltration membranes with pH dependent property prepared from poly (methacrylic acid) grafted polyethersulfone powder. J. Membr. Sci..

[167] Deng B., Li J., Hou Z., Yao S., Shi L., Liang G., Sheng K. (2008). Microfiltration membranes prepared from polyethersulfone powder grafted with acrylic acid by simultaneous irradiation and their pH dependence. Radiat. Phys. Chem..

[168] Mok S., Worsfold D., Fouda A., Matsuura T. (1994). Surface modification of polyethersulfone hollow-fiber membranes by γ-ray irradiation. J. Appl. Polym. Sci..

[169] Van der Bruggen B. (2009). Chemical modification of polyethersulfone nanofiltration membranes: A review. J. Appl. Polym. Sci..

[170] Schmidt M., Zahn S., Gehlhaar F., Prager A., Griebel J., Kahnt A., Knolle W., Konieczny R., Gläser R., Schulze A. (2021). Radiation-Induced Graft Immobilization (RIGI): Covalent Binding of Non-Vinyl Compounds on Polymer Membranes. Polymers.

[171] Adem E., Avalos-Borja M., Bucio E., Burillo G., Castillon F., Cota L. (2005). Surface characterization of binary grafting of AAc/NIPAAm onto poly (tetrafluoroethylene)(PTFE). Nucl. Instrum. Methods Phys. Res. Sect. B Beam Interact. Mater. At..

[172] Casimiro M., Leal J., Gil M. (2005). Characterisation of gamma irradiated chitosan/pHEMA membranes for biomedical purposes. Nucl. Instrum. Methods Phys. Res. Sect. B Beam Interact. Mater. At..

[173] He C., Gu Z. (2003). Studies on acrylic acid–grafted polyester fabrics by electron beam preirradiation method. I. Effects of process parameters on graft ratio and characterization of grafting products. J. Appl. Polym. Sci..

[174] Park J.S., Kim J.H., Nho Y.C., Kwon O.H. (1998). Antibacterial activities of acrylic acid-grafted polypropylene fabric and its metallic salt. J. Appl. Polym. Sci..

[175] Hassan M.S., Ibrahim H.M. (2016). Characterization and antimicrobial properties of metal complexes of polypropylene fibers grafted with acrylic acid using gamma irradiation. Polym. Adv. Technol..

[176] Montoya-Villegas K.A., Ramírez-Jiménez A., Licea-Claverie Á., Pérez-Sicairos S., Bucio E., Bernáldez-Sarabia J., Licea-Navarro A.F. (2019). Surface Modification of Polyester-Fabric with Hydrogels and Silver Nanoparticles: Photochemical Versus Gamma Irradiation Methods. Materials.

[177] Kumar V., Bhardwaj Y., Rawat K., Sabharwal S. (2005). Radiation-induced grafting of vinylbenzyltrimethylammonium chloride (VBT) onto cotton fabric and study of its anti-bacterial activities. Radiat. Phys. Chem..

[178] Flores-Rojas G., López-Saucedo F., Vázquez E., Hernández-Mecinas E., Huerta L., Cedillo G., Concheiro A., Alvarez-Lorenzo C., Bucio E. (2020). Synthesis of polyamide-6@ cellulose microfilms grafted with N-vinylcaprolactam using gamma-rays and loading of antimicrobial drugs. Cellulose.

[179] Huang K.-S., Yang C.-H., Huang S.-L., Chen C.-Y., Lu Y.-Y., Lin Y.-S. (2016). Recent advances in antimicrobial polymers: A mini-review. Int. J. Mol. Sci..

[180] Morais D.S., Guedes R.M., Lopes M.A. (2016). Antimicrobial approaches for textiles: From research to market. Materials.

[181] Seino S., Imoto Y., Kitagawa D., Kubo Y., Kosaka T., Kojima T., Nitani H., Nakagawa T., Yamamoto T.A. (2016). Radiochemical synthesis of silver nanoparticles onto textile fabrics and their antibacterial activity. J. Nucl. Sci. Technol..

[182] Ferraz C.C., Varca G.H., Ruiz J.-C., Lopes P.S., Mathor M.B., Lugão A.B., Bucio E. (2014). Radiation-grafting of thermo-and pH-responsive poly (N-vinylcaprolactam-co-acrylic acid) onto silicone rubber and polypropylene films for biomedical purposes. Radiat. Phys. Chem..

[183] Li X., Li P., Saravanan R., Basu A., Mishra B., Lim S.H., Su X., Tambyah P.A., Leong S.S.J. (2014). Antimicrobial functionalization of silicone surfaces with engineered short peptides having broad spectrum antimicrobial and salt-resistant properties. Acta Biomater..

[184] Goel N., Kumar V., Rao M., Bhardwaj Y., Sabharwal S. (2011). Functionalization of cotton fabrics by radiation induced grafting of quaternary salt to impart antibacterial property. Radiat. Phys. Chem..

[185] Yang J.M., Lin H.T., Wu T.H., Chen C.C. (2003). Wettability and antibacterial assessment of chitosan containing radiation-induced graft nonwoven fabric of polypropylene-g-acrylic acid. J. Appl. Polym. Sci..

[186] Terada A., Yuasa A., Tsuneda S., Hirata A., Katakai A., Tamada M. (2005). Elucidation of dominant effect on initial bacterial adhesion onto polymer surfaces prepared by radiation-induced graft polymerization. Colloids Surf. B. Biointerfaces.

[187] Murata H., Koepsel R.R., Matyjaszewski K., Russell A.J. (2007). Permanent, non-leaching antibacterial surfaces—2: How high density cationic surfaces kill bacterial cells. Biomaterials.

[188] Anjum N., Bellon-Fontaine M.N., Herry J.M., Riquet A.M. (2008). A novel process to develop modified polymeric surfaces for the analysis of bacterial adhesion: Surface properties and adhesion test. J. Appl. Polym. Sci..

[189] Huang C., Wang H., Xu Y.H. (2011). Functional finishing on silk fabric with acrylamide monomer and chitosan. Adv. Mater. Res..

[190] Ye F., Huang C., Jiang X., He W., Gao X., Ma L., Ao J., Xu L., Wang Z., Li Q. (2020). Reusable fibrous adsorbent prepared via Co-radiation induced graft polymerization for iodine adsorption. Ecotoxicol. Environ. Saf..

[191] Goel N., Rao M., Kumar V., Bhardwaj Y., Chaudhari C., Dubey K., Sabharwal S. (2009). Synthesis of antibacterial cotton fabric by radiation-induced grafting of [2-(Methacryloyloxy) ethyl] trimethylammonium chloride (MAETC) onto cotton. Radiat. Phys. Chem..

[192] Salmieri S., Khan R.A., Safrany A., Lacroix M. (2015). Gamma rays-induced 2-hydroxyethyl methacrylate graft copolymerization on methylcellulose-based films: Structure analysis and physicochemical properties. Ind. Crops Prod..

[193] Hoffman A. (1988). Radiation technology for immobilization of bioactive materials. IAEA Publication TECDOC-486.

[194] IAEA (1994). International Atomic Energy Agency, Radiation Processing Technology Applications in Bioengineering.

[195] IAEA (2002). Radiation Synthesis and Modification of Polymers for Biomedical Applications.

[196] IAEA (2015). Nanoscale Radiation Engineering of Advanced Materials for Potential Biomedical Applications.

[197] Peppas N.A., Hilt J.Z., Khademhosseini A., Langer R. (2006). Hydrogels in biology and medicine: From molecular principles to bionanotechnology. Adv. Mater..

[198] Anderson J.M., Rodriguez A., Chang D.T. (2008). Foreign body reaction to biomaterials. Semin. Immunol..

[199] Guimard N.K., Gomez N., Schmidt C.E. (2007). Conducting polymers in biomedical engineering. Prog. Polym. Sci..

[200] Balint R., Cassidy N.J., Cartmell S.H. (2014). Conductive polymers: Towards a smart biomaterial for tissue engineering. Acta Biomater..

[201] Ward M.A., Georgiou T.K. (2011). Thermoresponsive polymers for biomedical applications. Polymers.

[202] Ramanavičius A., Ramanavičienė A., Malinauskas A. (2006). Electrochemical sensors based on conducting polymer—Polypyrrole. Electrochim. Acta.

[203] Hasan J., Crawford R.J., Ivanova E.P. (2013). Antibacterial surfaces: The quest for a new generation of biomaterials. Trends Biotechnol..

[204] Webb R.C., Bonifas A.P., Behnaz A., Zhang Y., Yu K.J., Cheng H., Shi M., Bian Z., Liu Z., Kim Y.-S. (2013). Ultrathin conformal devices for precise and continuous thermal characterization of human skin. Nat. Mater..

[205] Zhang J., Peppas N.A. (2000). Synthesis and characterization of pH-and temperature-sensitive poly (methacrylic acid)/poly (N-isopropylacrylamide) interpenetrating polymeric networks. Macromolecules.

[206] Kingshott P., Wei J., Bagge-Ravn D., Gadegaard N., Gram L. (2003). Covalent attachment of poly (ethylene glycol) to surfaces, critical for reducing bacterial adhesion. Langmuir.

[207] Kobayashi M., Chang Y.-S., Oka M. (2005). A two year in vivo study of polyvinyl alcohol-hydrogel (PVA-H) artificial meniscus. Biomaterials.

[208] Chen C. (2017). Science mapping: A systematic review of the literature. J. Data Inf. Sci..

